# Highly Effective Biocides against *Pseudomonas aeruginosa* Reveal New Mechanistic Insights Across Gram-Negative Bacteria

**DOI:** 10.1021/acsinfecdis.4c00433

**Published:** 2024-10-23

**Authors:** Christian A. Sanchez, Germán G. Vargas-Cuebas, Marina E. Michaud, Ryan A. Allen, Kelly R. Morrison-Lewis, Shehreen Siddiqui, Kevin P. C. Minbiole, William M. Wuest

**Affiliations:** † Department of Chemistry, Emory University, Atlanta, Georgia 30322, United States; ‡ Department of Microbiology and Immunology, Emory University, Atlanta, Georgia 30322, United States; § Department of Chemistry, Villanova University, Villanova, Pennsylvania 19085, United States

**Keywords:** P. aeruginosa, disinfectant, antibiotic resistance, quaternary ammonium compounds, membrane, Gram-negative

## Abstract

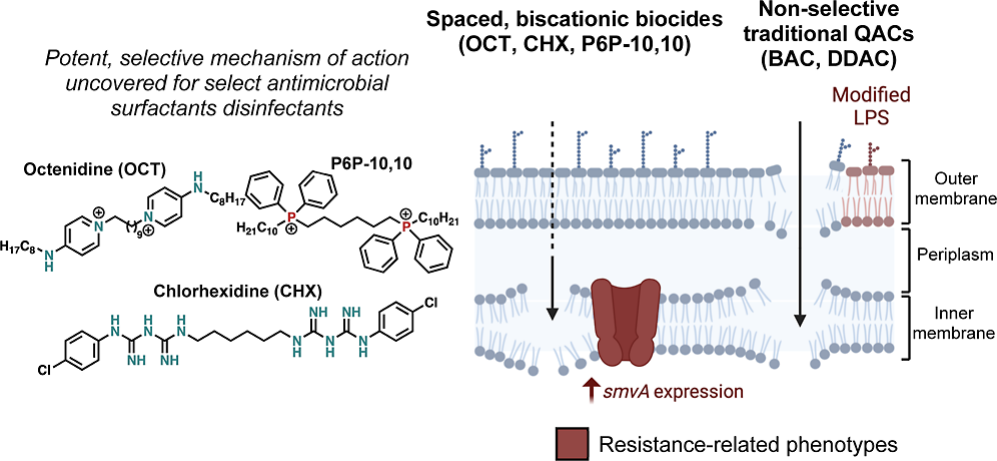

*Pseudomonas aeruginosa* is a major nosocomial pathogen that persists in healthcare settings despite rigorous disinfection protocols due to intrinsic mechanisms conferring resistance. We sought to systematically assess cationic biocide efficacy against this pathogen using a panel of multidrug-resistant *P. aeruginosa* clinical isolates. Our studies revealed widespread resistance to commercial cationic disinfectants that are the current standard of care, raising concerns about their efficacy. To address this shortcoming, we highlight a new class of quaternary phosphonium compounds that are highly effective against all members of the panel. To understand the difference in efficacy, mechanism of action studies were carried out, which identified a discrete inner-membrane selective target. Resistance selection studies implicated the SmvRA efflux system (a transcriptionally regulated, inner membrane-associated efflux system) as a major determinant of resistance. This system is also implicated in resistance to two commercial bolaamphiphile antiseptics, octenidine and chlorhexidine, which was further validated herein. In sum, this work highlights, for the first time, a discrete inner-membrane specific mechanism for the bolaamphiphile class of disinfectants that contrasts with the prevailing model of indiscriminate membrane interactions of commercial amphiphiles paving the way for future innovations in disinfectant research.

Cationic biocides are commonly found in antiseptic and disinfectant products that provide the first line of defense against microbial pathogens.^1,2^ Their utility expands from household and agricultural disinfection to biotic and abiotic surface decontamination in healthcare settings.^3^ Due to their widespread use, tens of millions of pounds of cationic biocides are produced in the United States annually for disinfection.^4^ Benzalkonium chloride and didecyldimethylammonium chloride are examples of highly effective cationic biocides used in healthcare settings due to their low toxicity and wide-spectrum efficacy. However, over decades of use, resistance to these biocides has increased at an alarming rate, threatening their utility.^5^ Biocide resistance has more recently been exacerbated by the COVID-19 pandemic, as stringent disinfection protocols became widespread and usage increased.^6^ Moreover, resistance to biocides has been shown to promote antibiotic resistance.^7,8^

Resistance to cationic biocides in Gram-negative bacteria is especially troubling due to the ever-shortening list of effective treatments against such pathogens. A major goal of this study was to evaluate the efficacy of both commercially available and our best-in-class cationic biocides against a panel of *Pseudomonas aeruginosa* clinical isolates. *P. aeruginosa* is an opportunistic Gram-negative pathogen that is responsible for over 500,000 deaths annually, is currently ranked as a Serious Threat by the CDC, and is a pathogen of Critical Priority by the WHO.^9^ Recently, Stribling et al. reported the decade-long persistence of *P. aeruginosa* strains in a hospital and how proper infection control was essential to suppress spread, highlighting the importance of effective disinfection protocols.^10^ There is a paucity of innovation in this field, and this lack of mechanistic nuance in the biocide mode of action has led to our current dire situation.

Cationic biocides generally act upon bacterial cells by binding to and subsequently disrupting the phospholipid cell membrane.^11^ With two cellular membranes, Gram-negative bacteria possess an added barrier to the uptake of disinfectants and other antimicrobials.^12^ Resistance to well-studied quaternary ammonium compound (QAC) disinfectants in Gram-negative bacteria includes the expression of efflux pumps, upregulation of polyamines like spermidine, membrane lipid changes, and even biodegradation.^13^ These mechanisms typically confer cross-resistance against other QACs leading to widespread resistance for these biocides. Illustrating this point, we recently reported the identification of a clinical isolate of *Acinetobacter baumannii* that is resistant to most, if not all, commercially available classes of QACs.^14^ These findings suggest the presence of underlying mechanistic subtleties and underscore the importance of understanding how Gram-negative bacteria can develop resistance to these biocides.

As detailed below, we conducted a screen against a panel of multidrug-resistant *P. aeruginosa*clinical isolates and observed broad resistance to QAC biocides but, excitingly, superior efficacy of our novel quaternary phosphonium compounds (QPCs). While exploring the bactericidal and resistance mechanisms of QPC P6P-10,10, we were led to uncover distinct, structurally predictable mechanistic determinants of cationic biocides in Gram-negative bacteria that have profound implications for disinfectant resistance mechanisms. Through this work, we have elucidated how the chemical properties of cationic biocides influence the specificity of membrane targets in a panel of high-priority Gram-negative pathogens, and in turn, how this is reflected in their antimicrobial activity. These findings provide insight for rational design of cationic biocides against Gram-negative bacteria.

## Results

### *P. aeruginosa* Clinical Isolates are Broadly Cationic Biocide Resistant

**Figure 1:**
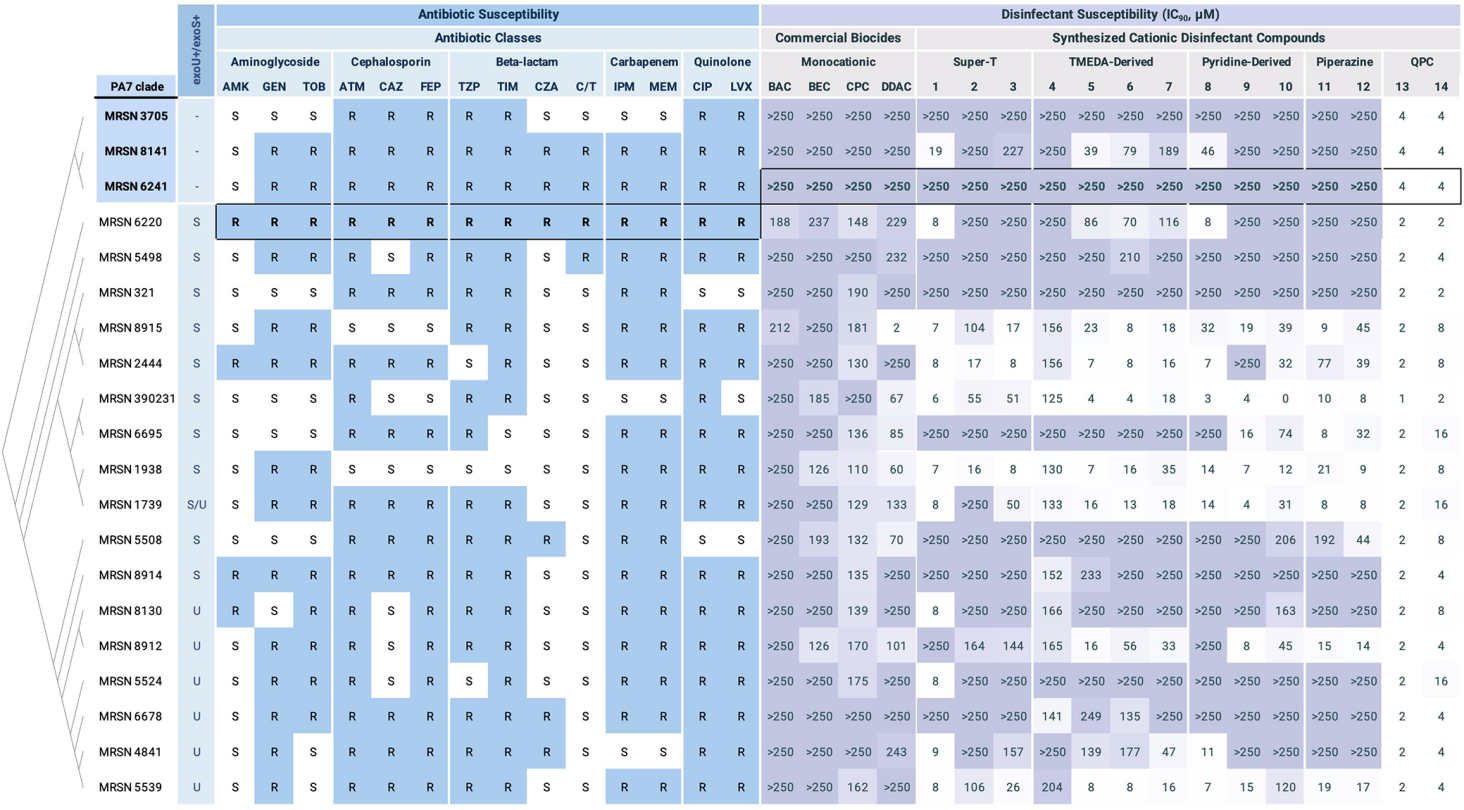
Susceptibility of the *P. aeruginosa* clinical isolates to a panel of 14 antibiotics, 4 commercial QACs, and 14 of our previously reported cationic disinfectant compounds. For the structures of cationic biocides 1–14, see Supporting Information. The listed antibiotic susceptibilities were previously reported by Lebreton et al., wherein resistance (*R*) or susceptibility (*S*) was determined according to CLSI guidelines. Antimicrobials are grouped by drug class, and their susceptibilities are mapped against the phylogeny of the clinical isolates, generated using RAxML from alignment of the core genomes. Antibiotic abbreviations: AMK, amikacin; GEN, gentamicin; TOB, tobramycin; ATM, aztreonam; CAZ, ceftazidime; FEP, cefepime; TZP, piperacillin/tazobactam; TIM, ticarcillin/clavulanic acid; CZA, ceftazidime/avibactam; C/T, ceftolozane/tazobactam; IPM, imipenem; MEM, Meropenem; CIP, ciprofloxacin; LVX, levofloxacin. Cationic biocides abbreviations: BAC, benzalkonium chloride, BEC, benzethonium chloride, CPC, cetylpyridinium chloride, DDAC, didecyldimethylammonium chloride.

We sought to interrogate the efficacy of cationic biocides against a panel of *P. aeruginosa* clinical isolates from the Multidrug-Resistant Organism Repository and Surveillance Network (MRSN).^15^ This panel was originally designed to maximize genetic diversity of *P. aeruginosa* strains, but it also provides a diverse range of antibiotic resistance phenotypes. Thus, the panel provides an excellent avenue to study antimicrobial resistance in this bacterial species. We selected a subset of 20 genetically diverse multiple drug-resistant, extensively drug-resistant, and pan-drug-resistant members, and collected IC_90_ values for four commercial disinfectants, 12 of our best-in-class QACs, and two of our best QPCs. Initially, we determined MICs for each cationic biocide listed and observed superior antimicrobial activity of our QPCs (Figure S1). However, we were met with trailing growth for certain QACs in different isolate strains, corresponding with a heteroresistance phenotype like our previous observations in *A. baumannii*.^16^ Due to the presence of resistant subpopulations above the MIC, we used IC_90_ values as a proxy for disinfectant efficacy to standardize our results (Figure 1). Across the panel, we observed a high degree of cationic biocide resistance in both commercially available QACs and our next-generation QACs, compared to previously reported results with laboratory strain PAO1 and our screen with *A. baumannii*.^16^ We observed no apparent correlation between antibiotic resistance and disinfectant resistance, but we found each of the PA7-related species displayed a high degree of disinfectant resistance. The PA7 clade is a taxonomic outlier that possess an extended resistance spectrum and typically possesses an increased biofilm forming character.^17^ To our knowledge, this represents the first work demonstrating a potential connection between disinfectant resistance and the PA7 clade.

### Next Generation QPCs are Effective against Highly Antibiotic-Resistant *P. aeruginosa* Strains

Additionally, we observed the superior inhibitory efficacy of QPCs P6P-10,10 and P6P-12A,12A (**13** and **14** in Figure 1, respectively) where traditional nitrogen-centered disinfectants fell short. Whereas many QACs displayed trailing growth obscuring the MIC, QPCs P6P-10,10 and P6P-12A,12A had distinct MICs averaging in the single-digit micromolar range across the panel. To understand the ability of QPCs to overcome disinfectant resistance mechanisms, we sought to further investigate any mechanistic differences that might be present.

### Outer Membrane of *P. aeruginosa* is Not Appreciably Influenced by the Presence of P6P-10,10

**Figure 2:**
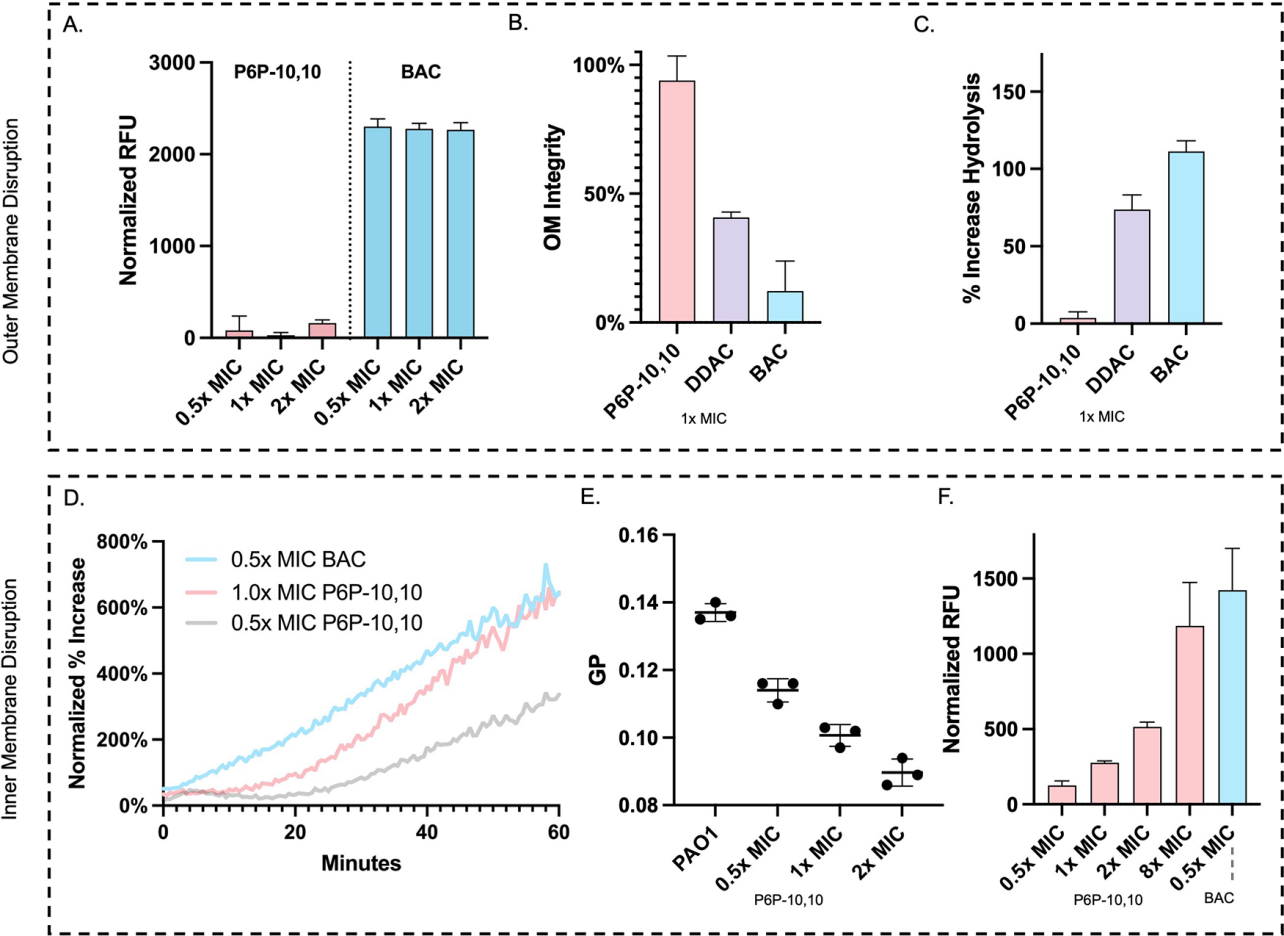
Evaluation of cationic biocide activity on the outer and inner membrane of *P. aeruginosa* PAO1. (A) NPN uptake was assessed fluorometrically to measure outer membrane perturbation with increasing concentrations of BAC and P6P-10,10. (B) Lysozyme permeability assay to assess outer membrane integrity through OD_600_ measurements upon lysozyme and disinfectant treatment. (C) Effect of disinfectant treatment on nitrocefin hydrolysis mediated by outer membrane disruption. (D) Inner membrane depolarization measured by DiSC_3_-(5) upon QAC and QPC treatment. (E) Laurdan generalized polarization (GP) to assess inner membrane fluidity, where lower values indicate increases in fluidity. (F) Propidium iodide cytoplasmic entry as a measure of inner membrane disruption.

We used various membrane disruption assays to study the effects of P6P-10,10 on the outer membrane of *P. aeruginosa* lab strain PAO1.^18−21^ Starting with the *N*-phenyl-1-naphthylamine (NPN) uptake assay, we were surprised to see that there was minimal uptake of NPN induced by P6P-10,10 treatment when compared to commercial QAC benzalkonium chloride (BAC) at increasing concentrations (Figure 2A). No significant changes were observed upon treatment with higher BAC concentrations, suggesting that BAC saturates the outer membrane well below the MIC. Upon dosing P6P-10,10 at sequentially higher concentrations above the MIC, we observed a dose-dependent increase in NPN uptake, but it still was substantially less than the other known membrane disrupters (Figure S2A). Testing at a lower cell density (OD_600_ = 0.05), we observed similar results (Figure S2B). To further investigate the effect of P6P-10,10 on the outer membrane, we used a lysozyme permeability assay to test the effects of biocide at the MIC. Again, we observed that P6P-10,10 exerts no appreciable permeabilizing effect on the outer membrane of *P. aeruginosa*. However, QACs BAC and dodecyl dimethylammonium chloride (DDAC) displayed potent membrane permeabilizing effects (Figure 2B), suggesting distinct mechanisms of action between these cationic biocides. Additionally, a nitrocefin hydrolysis assay supported the previous results and indicated that P6P-10,10 exerts a minimal effect on the outer membrane of *P. aeruginosa* at the MIC (Figure 2C).

### P6P-10,10 Selectively Targets the Inner Membrane of *P. aeruginosa*

To understand the effects of the QPC on the inner membrane, we used a 3,3′-dipropylthiadicarbocyanine iodide [DiSC_3_-(5)] membrane depolarization procedure to measure inner membrane disruption at a low cell density (OD_600_ = 0.05).^22^ P6P-10,10 displayed appreciable depolarization at 0.5× and 1× MIC comparable to BAC (Figure 2D). Furthermore, by using a Laurdan generalized polarization (GP) assay, we observed similar dose-dependent responses.^23^ A dose-dependent decrease in GP upon P6P-10,10 treatment was observed, which correlates to an increase in membrane fluidity consistent with an inner membrane disruption mechanism (Figure 2E). Additionally, P6P-10,10 dose-dependently induced membrane disruption, as determined by increase in fluorescence of the dye propidium iodide (Figure 2F).^24^ The higher concentration of QPC required to induce these membrane perturbations compared to the DiSC3-(5) assay is due to a sizable inoculum effect of P6P-10,10 on PAO1, where a significant increase in MIC was observed when a larger inoculum was used (Figure S3). These inner membrane disruption assays suggest that, while P6P-10,10 has no appreciable effect on the outer membrane, it possesses an inner membrane-specific mechanism of action, distinct from QACs BAC and DDAC. To the best of our knowledge, this is the first specific chemotype that is selective for the inner membrane of Gram-negative bacteria and may shed light on a new mechanism of action for the future design of disinfectants.

### Antagonism Assays Support an Inner Membrane-Specific Mechanism of Action

To further explore the inner membrane specificity of P6P-10,10, we performed two different antagonism assays designed to probe this putative mechanism of action. Spermidine (Spd) is a cationic polyamine of Gram-negative bacteria. In *P. aeruginosa*, spermidine has been shown to localize to and protect the outer membrane from antibiotic treatment.^25,26^ In addition, Kwon et al. demonstrated that addition of exogenous spermidine antagonizes activity of the cationic membrane disrupter polymyxin B.^27^ Furthermore, an increase in spermidine production is a known transcriptional response to treatment with BAC, presumably because it masks the negative potential on the exterior of the cell.^13^ To probe the mechanism of our lead QPC, *P. aeruginosa* was treated with BAC, DDAC, and P6P-10,10 in the presence of 5 mM Spd. We hypothesized that if BAC and DDAC target the outer membrane but P6P-10,10 does not, then we would observe Spd antagonism with BAC and DDAC but not against P6P-10,10. As hypothesized, we observed that exogenous Spd antagonized BAC and DDAC, as evidenced by the increase in MIC (Figure S4). However, the MIC of P6P-10,10 remained unchanged, suggesting that the outer membrane is not the target of P6P-10,10. The second antagonism assay involved the use of the protonophore carbonyl cyanide *m*-chlorophenyl hydrazone (CCCP). CCCP decouples the electrical potential of the cytoplasmic membrane, and dosing at subinhibitory concentrations has shown to be advantageous to probe systems related to the inner membrane of bacteria.^28,29^ Previous results while exploring P6P-10,10 in *P. aeruginosa* have illustrated that CCCP is antagonistic to QPC treatment.^30^ We replicated this previous experiment and demonstrated that CCCP is antagonistic to P6P-10,10, while having no profound effect on BAC treatment under the experimental conditions tested (Figure S4). Taken together, these results further support the inner membrane specificity of P6P-10,10 activity.

### Distinct Mechanisms of Resistance to Cationic Biocides

**Figure 3:**
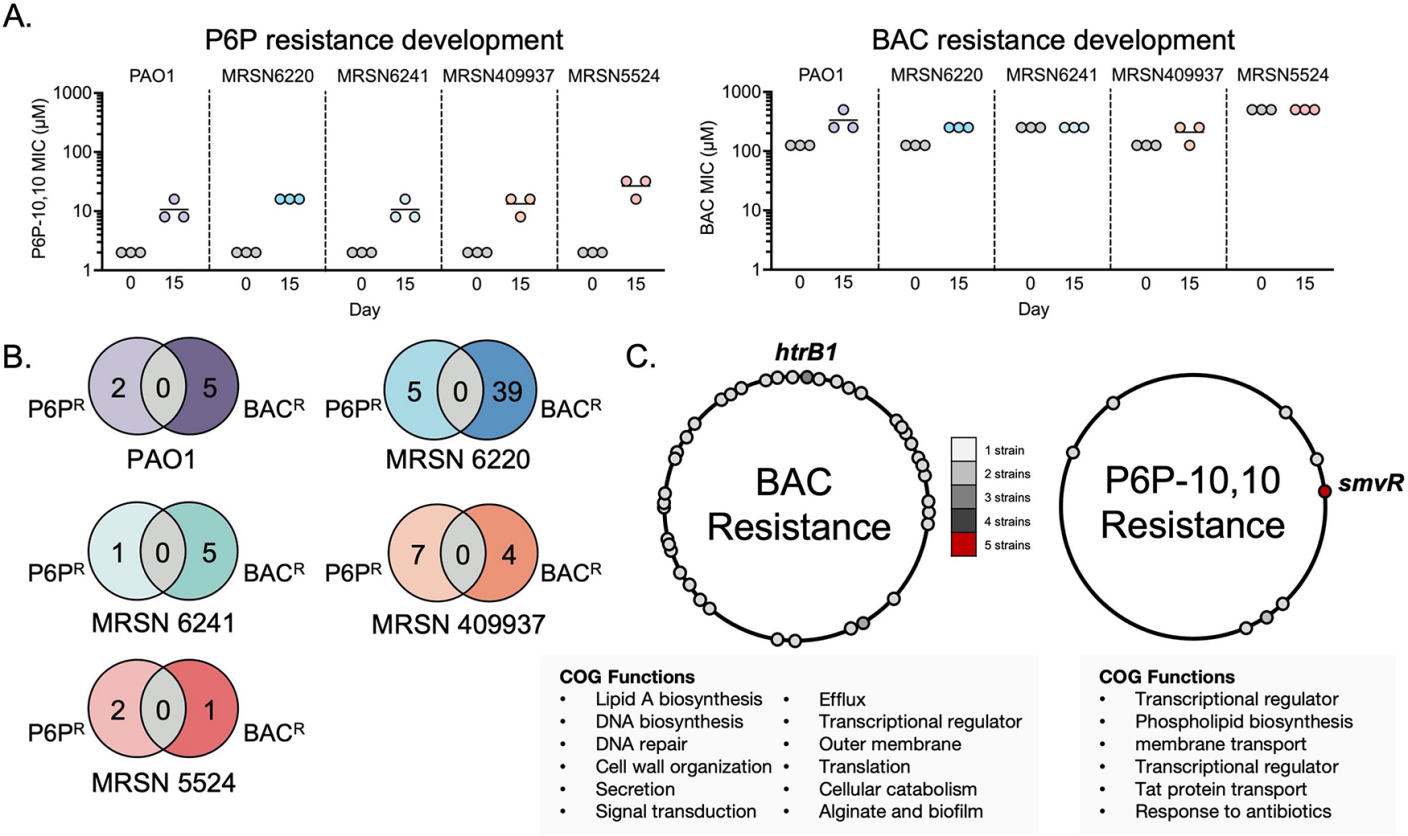
Distinct cationic biocide resistance profiles in response to P6P-10,10 and BAC. (A) MIC values for P6P-10,10 (left) and BAC (right) before (day 0) and after completion of resistance selection assay (day 15). Three independent biological replicates were performed per strain. (B) Venn diagrams of genetic variants identified in P6P- and BAC-resistant mutants of 5 *P. aeruginosa* strains: PAO1, MRSN6220, MRSN6241, MRSN409937 and MRSN5524. (C) Schematic of genomic location of genes with identified genetic variations associated with resistance to P6P-10,10 and BAC. Genes were mapped to *P. aeruginosa* PAO1 reference genome for simplicity. Cluster of orthologous genes (COG) cellular functions of genes with mutations identified in adaptation to each cationic biocide is reported.

We hypothesized that since these cationic biocides display distinct modes of action, resistance to these compounds would be achieved through distinct mechanisms. In a recent study, distinct intrinsic mechanisms of resistance were identified in *A. baumannii* using transposon-directed insertion-site sequencing (TraDIS) and a panel of 10 biocides.^31^ To explore acquired resistance mechanisms to cationic biocides, we performed a resistance selection assay by exposing the reference *P. aeruginosa* PAO1 strain and four additional clinical isolates with different MDR profiles to subinhibitory concentrations of BAC and P6P-10,10 over a period of 15 days, and isolated mutants with stable increases in MIC for both cationic biocides (Figure 3A). One resistant mutant from each genetic background was selected and subjected to whole-genome sequencing to identify resistance determinants associated with decreased susceptibility to these biocides. We observed no overlap in resistance mutations to these cationic biocides, correlating with these biocides having a distinct mode of action (Figure 3B). In addition, while adaptation to P6P-10,10 can be pinpointed to a few genetic loci, BAC adaptation is associated with mutations in multiple genetic loci across the genome (Figure 3C). Loss of function mutations in *htrB1* were frequently identified in the BAC-resistant mutants (3/5 strains) in addition to mutations in genes associated with several cellular functions including DNA replication and repair, biofilm production, and virulence, among others (Figure 3C). In contrast, loss of function mutations in *smvR*, the negative transcriptional regulator of SmvA, were identified in all P6P-resistant mutants. Some BAC-resistant mutants show a small increase in P6P-10,10 MIC, but the converse was not observed (Table S4). Mutations in smvR have been implicated in chlorhexidine and octenidine resistance. These data suggest that SmvA is a major bolaamphiphile resistance determinant in *P. aeruginosa*. Adaptations to cationic biocides with distinct mechanisms of action result in completely different resistance profiles in *P. aeruginosa*.

### SmvA is a Major P6P-10,10 Resistance Determinant

**Figure 4:**
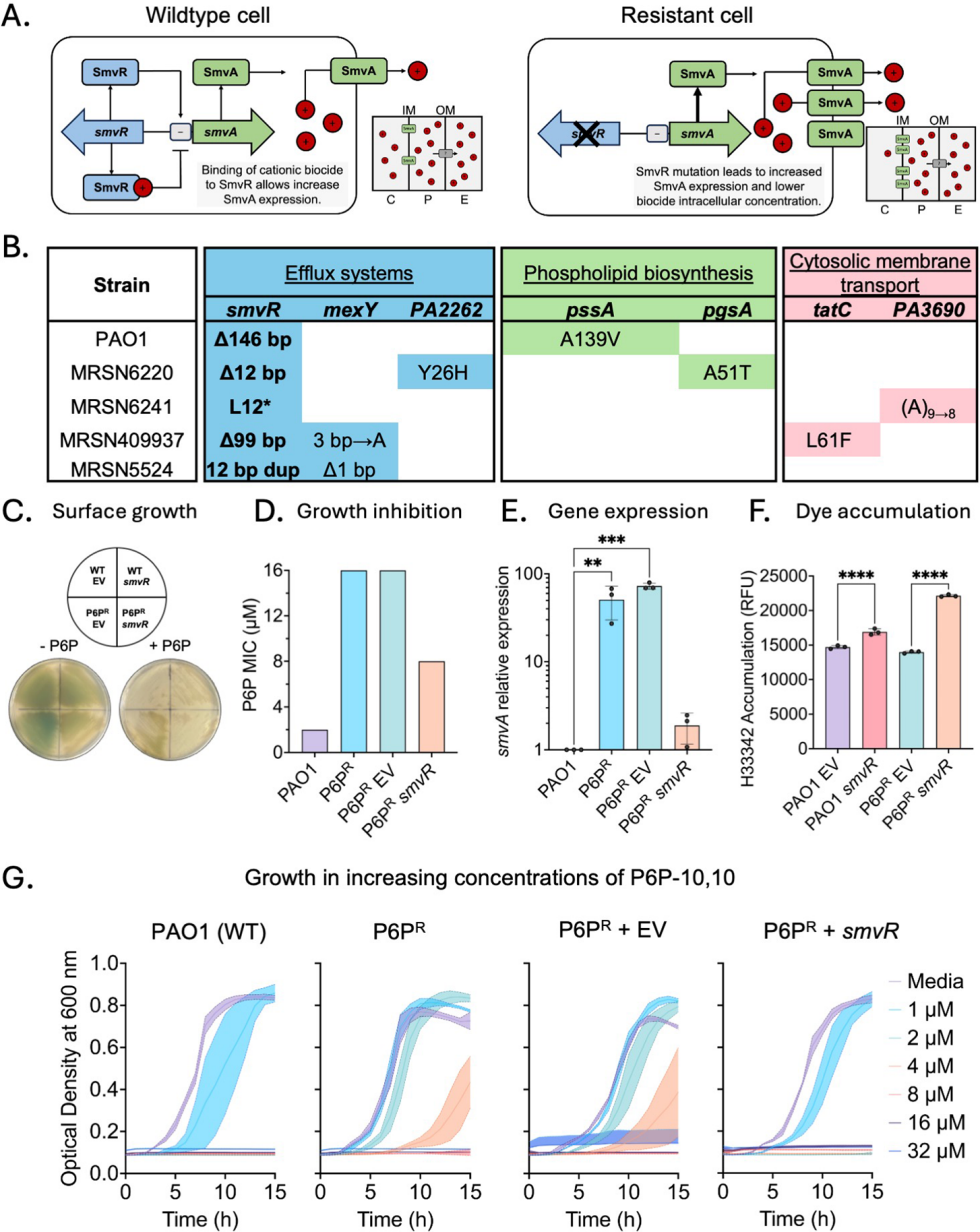
The efflux system SmvRA is a major resistance determinant of the QPC, P6P-10,10. (A) Schematic of regulation of SmvA efflux pump expression (C, cytoplasm; P, periplasm; E, environment; IM, inner membrane; OM, outer membrane). (B) Mutations identified associated with P6P resistance in the five *P. aeruginosa* genetic backgrounds. (C) *P. aeruginosa* PAO1 (WT) and P6P-resistant (P6P^R^) strains with either empty vector (pUCP30T) or vector containing a functional copy of *smvR* with its native promoter (pUCP30T-*smvR*) incubated in LB plates with and without P6P-10,10 (700 μg). (D) MIC values of PAO1 (WT) and P6P-resistant (P6P^R^) strains with either empty vector (pUCP30T) or vector containing a functional copy of *smvR*. (E) RT-qPCR expression analysis of *smvA*. (F) Hoechst 33342 accumulation assay in strains with either empty vector (pUCP30T) or vector containing a functional copy of *smvR*. (G) Growth curves of *P. aeruginosa* PAO1 (WT) and P6P-resistant (P6P^R^) strains with either empty vector (pUCP30T) or vector containing a functional copy of *smvR* in the presence of increasing concentrations of P6P-10,10. The mean and standard deviation (SD) of 6 replicates is plotted. RT-qPCR and dye accumulation was analyzed using one-way ANOVA with Tukey correction for multiple comparisons. **, *p*-value ≤0.01; ***, *p*-value ≤0.001; ****, *p*-value ≤0.0001.

SmvR is a Tet-like repressor of SmvA, a major facilitator superfamily (MFS) efflux pump that has been shown to provide resistance to other cationic biocides. Derepression of SmvA can occur by binding of the cationic compound to SmvR, causing a conformational change that prevents it from effectively binding to DNA, or by loss of function mutations in *smvR* (Figure 4A). With 14 transmembrane domains, SmvA is classified within the drug/proton antiporter (DHA) DHA2 member alongside QAC efflux pump (QacA) and LfrA, likely sharing an “asymmetric rocker-switch” motion coordinated by extracellular loops.^32,33^ Interestingly, a loss of function mutation in *smvR* was identified in strains resistant to P6P-10,10 in all genetic backgrounds (Figure 4B). To assess the role of the SmvRA system in P6P-10,10 resistance, we performed genetic complementation test by introducing a wildtype SmvR copy with its predicted native promoter in pUCP30T vector to restore repression of SmvA, and measured susceptibility to P6P (Figure 4C).^34^ Restoration of SmvR function partially restored susceptibility to P6P-10,10 as measured by MIC assays (Figure 4D). In addition, SmvR complementation restores expression of SmvA close to wildtype levels, as measured by RT-qPCR (Figure 4E). This also correlated with an increased accumulation rate of Hoechst 33342, used as a proxy for efflux activity (Figure 4F). To further corroborate the involvement of SmvRA in P6P-10,10 resistance, we exposed the wildtype and P6P-10,10-resistant strains harboring either pUCP30T with a functional copy of SmvR or the empty vector as control to increasing concentrations of P6P-10,10 and observed that introduction of a functional copy of SmvR led to a similar cationic biocide susceptibility profile as wildtype *P. aeruginosa* PAO1 (Figure 4G). In sum, these data unveil SmvA as a key resistance determinant to the novel P6P-10,10 and highlight the importance of this MFS efflux system in cationic biocide resistance. Further, these data unveil different mutations associated with distinct modes of action for cationic biocides.

### Cationic Biocides Possess Distinct Mechanisms in Gram-Negative Bacteria That are Structurally Predictable

**Figure 5:**
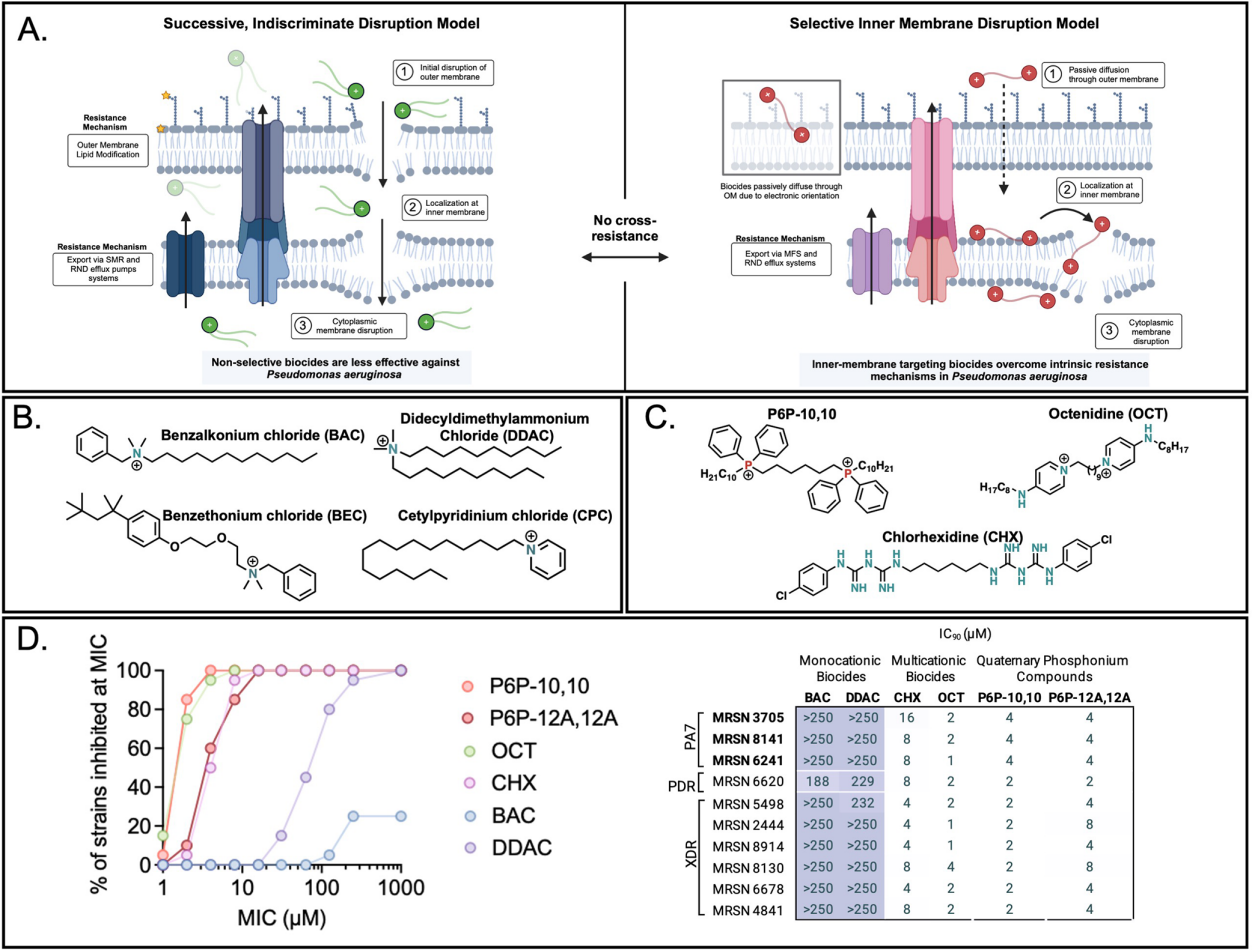
Mechanistic insights of cationic biocide classes from this study. (A) Mechanistic proposal of spaced-cationic biocides contrasted with traditional QACs BAC and DDAC, whereby spaced-cationic biocides with a spacer selectively disrupt the inner membrane while QACs show nonselective disruption of both outer and inner membranes. (B) Structures of disinfectants with spaced-cationic structures in this study: P6P-10,10, octenidine, and chlorhexidine. (C) Structures of QACs BAC and DDAC. (D) CHX and OCT possesses comparable activity to P6P-10,10 and P6P-12A,12A against a panel of 20 clinical isolates of *P. aeruginosa*. Abbreviations: PDR, pan-drug resistant (resistant to all 14 agents tested by Lebreton et al.); XDR, extensively drug resistant (defined as nonsusceptible to ≥1 agent in all but <2 categories).

Due to the shared resistance determinant among OCT, CHX, and P6P-10,10 in Gram-negative species, we hypothesized that they share an inner membrane-specific mechanism of action in *P. aeruginosa*.^23,35,36^ Using the NPN uptake assay for outer membrane permeabilization, we observed no appreciable NPN uptake upon treatment with OCT or CHX when compared to BAC and DDAC, like P6P-10,10 (Figure S2C). From the presence of *smvR* mutations in other Gram-negative species, we hypothesized that this inner membrane-specific mechanism of action was not limited to *P. aeruginosa*. To test our hypothesis, we assessed the mechanism of action of P6P-10,10, OCT, and CHX in additional Gram-negative species *Escherichia coli* MC4100 and *A. baumannii* ATCC 19606. NPN uptake assays revealed that P6P-10,10, OCT, and CHX do not appreciably perturb the outer membrane in any of the species tested (Figure S5). Importantly, this demonstrates that cationic biocides act via different mechanisms in Gram-negative bacteria and uncovers a mechanistic difference that was previously unknown. This was additionally validated by antagonism experiments with antibiotics with reduced uptake in Gram-negative species in combination with inner membrane specific biocides (Figure S7). With this new knowledge in hand, we revisited our initial *P. aeruginosa* panel and tested CHX and OCT against the 20 clinical isolates. For these multicationic disinfectants, we observed potency like that of the QPCs tested (Figures 5 and S4).

Through examining the molecular structures between the P6P-10,10, OCT, and CHX compared to QACs BAC and DDAC, we observed the presence of spatially separated, charged moieties in the former three. These compounds possess cationic warheads all separated by 6–8 methylene moieties reminiscent of bolaamphiphiles. We posit that by possessing these cationic moieties separated by a linker, these biocides selectively disrupt the inner membrane of Gram-negative bacteria without producing a strong effect on the outer membrane. Future studies will further probe this phenomenon to better understand how the chemical composition of each membrane leads to its chemo-selectivity.

## Discussion

A longstanding problem in medicinal chemistry is the challenge associated with targeting specific biological targets and cellular components over others. Here we report that cationic antimicrobials P6P-10,10, OCT, and CHX, display the ability to passively diffuse through the outer membrane of Gram-negative bacteria and target the cytoplasmic membrane. Certain bolaamphiphiles are known to penetrate membranes without disruption^37−39^ while others have been optimized for membrane-disrupting activity.^40−42^ Here, we observe bolaamphiphiles that nondisruptively penetrate the bacterial outer membrane and selectively perturb the cytoplasmic membrane, providing a chemical basis for cytoplasmic membrane targeting in Gram-negative bacteria. These results were further supported by antagonism assays performed with Spd and CCCP. A change in MIC was observed for DDAC upon CCCP treatment. This highlights the continuum that exists among membrane active disinfectants in terms of membrane selectivity as seen with the NPN data (Figure S2C). DDAC appears to have a slight preference toward the inner membrane while still being nonselective. It has been noted that the segmented amphiphilicity of the bolaamphiphiles endow this class of molecules with complex self-assembly behavior.^43^ We hypothesize that this supramolecular structure facilitates passive diffusion through the outer membrane of Gram-negative bacteria. The opposing polar heads separated by a sufficiently long alkyl chain have been proposed to adopt a U-shape (in the outer leaflet) and transmembrane conformations in the lipid bilayer of the bacterial cytoplasmic membrane.^44^ Molecular dynamics studies of mono- and multicationic QACs have suggested an alternative mechanism of membrane integration and disruption in a one-two fashion of electrostatic attraction and subsequent membrane penetration and disruption.^45^ This work uncovers that the mechanistic differences of cationic disinfectants expand beyond lipid bilayer interactions, providing a framework for cationic biocide selectivity determination.

Despite having a primarily external target in bacteria, certain cationic biocides—almost counterintuitively—possess an internally regulated resistance mechanism: transcriptional flux of efflux pump expression. In Gram-positive species, QacA is a classic example of an efflux pump that confers resistance against cationic species.^46,47^ Interestingly, QacA is regulated by the negative transcriptional regulator QacR, which requires the binding of substrate before it is released from DNA to allow transcription of the downstream gene *qacA*.^48^ In the current work, we observe an analogous system with SmvRA conferring resistance against bolaamphiphilic disinfectants in *P. aeruginosa*. Since SmvA is in the cytoplasmic membrane, these cationic species are only exported from the cytoplasm to the periplasm. Efflux from the cytoplasm may be sufficient to mitigate the harmful effects of the biocides or additional export mechanisms such as passive diffusion out of the cell or some other unknown export process may exist. Requiring intracellular accumulation of amphiphile (though presumably at very low concentrations) to induce resistance mechanisms suggests that internal effects of the disinfectant are not negligible. This highlights how the linear mechanism of action that is presented is simply the sum average of all the interactions occurring between the lipid membranes and the cationic amphiphiles.

From this perspective, the observed inner membrane selectivity is likely the result of a high degree of localization at the inner membrane and unfavorable on–off binding kinetics between the outer membrane and the bolaamphiphiles. Furthermore, the diffuse charge of the phosphorus cation or the delocalized, lipophilic ammonium cations could contribute to this effect. In contrast, nonbolaamphiphile disinfectants interact strongly with the outer membrane, leading to disruption. This effect can be amplified by increasing the affinity between the membrane and the amphiphile by adding cationic centers, as evidenced by the potency of multicationic QACs. While multicationic QACs are effective against a range of antimicrobial-resistant pathogens, results from initial screen display the superiority of the bolaamphiphiles against highly resistant biocide resistant clinical isolates. Importantly, we demonstrate how the distinct mechanism of action of spaced-cationic biocides can overcome resistance to traditional QACs in Gram-negative bacterial species *P. aeruginosa*, which is notoriously difficult to eradicate through biocide treatment.^31^

*P. aeruginosa* is an opportunistic pathogen that possesses a range of complicated clinical manifestations, especially in patients with cystic fibrosis.^9,49^ This pathogen has received the highest risk level by both the World Health Organization and the CDC due to its propensity to develop antimicrobial resistance with almost a third of all isolates of Europe demonstrating resistance to at least one antimicrobial treatment group.^50−52^ Recently, Mc Gann et al. reported a case of a wounded soldier coinfected with six bacterial strains, three of which were extremely drug-resistant *P. aeruginosa* strains, highlighting the danger this pathogen poses during conflicts.^53^

Cationic biocides, such as QACs, are an important part of disinfection protocols in the healthcare and food industries as a barrier to prevent the spread of this pathogen and others. This widespread use of biocides across industrial settings has led to increased resistance as a consequence.^6^ As alternative compounds to the overused QACs, our group has developed a series of QPCs that show improved activity against a diverse group of pathogenic bacteria.^30,54−60^ The effectiveness of one such compound, P6P-10,10, against *A. baumannii* strains displaying high levels of resistance to diverse cationic biocides suggested that P6P-10,10 might possess a distinct mode of action compared to common QACs.^12^

In this work, we investigated the mechanistic nuances of cationic biocide antimicrobial activity by comparing the QPC P6P-10,10 to commercially available QACs. We tested these disinfectant compounds against a panel of highly antibiotic-resistant *P. aeruginosa* clinical isolates, and, as anticipated, observed high levels of resistance to a wide range of biocides (Figure 1). Strikingly, the QPCs maintained strong antimicrobial activity against all the isolates, suggesting that these compounds evade preexisting mechanisms of antibiotic and biocide resistance. Biocides are believed to have multiple cellular targets, with membrane disruption being the primary mechanism of action.^61^ While BAC and DDAC showed potent outer membrane disruption, P6P-10,10 minimally affected the outer membrane. In contrast, when the integrity of the inner membrane was assessed, P6P-10,10 exhibited strong and dose-dependent disruption, suggesting that P6P-10,10 preferentially targets the inner membrane in *P. aeruginosa*.

We have also shown that increased expression of the MFS efflux pump SmvA is the main resistance mechanism to the QPC, P6P-10,10, with all P6P-10,10-resistant strains having a loss of function mutation in its negative regulator SmvR (Figure 4). Genetic complementation studies indicated that, while SmvA is the predominant resistance determinant for P6P-10,10, complete repression of SmvA expression or mutations in other genes (e.g., *pssA*) are likely required for full complementation and return susceptibility to wildtype levels (Figure 4). Importantly, SmvA is also associated with resistance to octenidine and chlorhexidine, two bolaamphiphiles that share a preference to disrupt the inner membrane.^23,35,36^ These results provide a link between chemical nature of these cationic biocides, their cellular target, and the determinants associated with resistance.

In summary, this study uncovers several key mechanistic nuances of how different cationic biocides exert their antimicrobial effects on Gram-negative bacteria, how their structural properties can provide insights into their mechanism of action, and how this information can be used to predict resistance determinants that decrease susceptibility to these compounds.

## Materials and Methods

### Bacterial Strains and Growth Conditions

All strains and plasmids are listed in Table S1. Bacterial strains were streaked onto lysogeny broth (LB) agar (Sigma-Aldrich, 1102830500) plates and incubated (NuAire, Plymouth, MN) at 37 °C overnight. Liquid cultures were inoculated with single colonies from plates and incubated for 18–24 h at 37 °C with shaking. Media was supplemented with gentamicin (30–60 μg/mL) for vector maintenance in *P. aeruginosa* strains, as needed. *P. aeruginosa* clinical isolates were obtained from the Multidrug-Resistant Organism Repository and Surveillance Network (MRSN).

Growth curves were performed in 96-well flat-bottom plates (Falcon, 351172) with shaking. Optimal density was measured at a wavelength of 600 nm (OD_600_) every 10 min and growth was monitored over 24 h. OD_600_ measurements were obtained using a SpectraMax iD3 plate reader (Molecular Devices, United States). Growth curve experiments were performed on different days with independent biological replicates with at least 3 technical replicates per strain/condition.

### Minimum Inhibitory Concentration (MIC) Assays

To determine the MIC values, compounds were serially diluted 2-fold from stock solutions (1.0 mM) to yield 12,100 μL test concentrations, wherein the starting concentration of DMSO was 2.5%. Overnight cultures of each strain were diluted to ca. 10^6^ cfu/mL in MHB and regrown to midexponential phase, as determined by optical density recorded at 600 nm (OD_600_). All cultures were then diluted again to ca. 10^6^ cfu/mL and 100 μL were inoculated into each well of a U-bottom 96-well plate (Falcon, 351177) containing 100 μL of compound solution. Plates were incubated statically at 37 °C for 72 h upon which wells were evaluated visually for bacterial growth. The MIC was determined as the lowest concentration of compound resulting in no bacterial growth visible to the naked eye, based on the highest value in three independent experiments. Aqueous DMSO controls were conducted for each strain. Strains of *Staphylococcus aureus* MSSA (SH1000), *E. coli* (MC4100), *P. aeruginosa* (PAO1), *A. baumannii* (ATCC 17978), CA-MRSA (USA300-0114), and HA-MRSA (ATCC 33591) were grown with shaking at 37 °C overnight from freezer stocks in 5 mL of the indicated media: SH1000, MC4100, USA300–0114, and PAO1 were grown in BD Mueller–Hinton broth (MHB), whereas ATCC 33591 was grown in BD tryptic soy broth (TSB). MRSN isolates were grown in MHB in the same fashion as previously described.

### Calculation of IC_90_ Values

To determine the IC90 values for each compound against the clinical isolates, the OD_600_ for each compound concentration against each strain was recorded. Using Prism 9 (GraphPad software, v. 9.3.1), the IC90 values for each disinfectant compound against each strain were calculated. The OD_600_ measurements were used as inputs, then normalized to fit 0% (equal to media blank) to 100% (maximum OD_600_ for each strain). The analysis was then performed on the normalized data using the dose–response model with a least-squares regression fit, wherein outliers (*Q* = 1%) were excluded and no weighting method was applied.

### NPN Uptake Assay

*P. aeruginosa* PAO1 were grown overnight in LB, then regrown from a 1:100 dilution in fresh media for 5 h to an OD_600_ of 0.500. Cells were harvested by centrifugation (4000 rpm, 25 °C, 10 min), washed twice with assay buffer (5 mM HEPES, 5 mM glucose, pH 7.2), and resuspended in assay buffer to a final OD_600_ of 1. Then, 100 μL of washed cells and 100 μL of assay buffer containing 20 μM NPN were together and incubated for 10–30 min 198 μL of cells and NPN added to a 96-well optical-bottom black plate. Either 2 μL of a chemical compound or the corresponding solvent was added to each well, and fluorescence was immediately monitored at an excitation wavelength of 350 nm and an emission wavelength of 420 nm for 7 min at 30 s intervals.
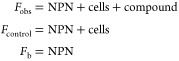

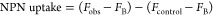


Twenty μM NPN in assay buffer was made from a 5 mM stock of NPN in acetone.

### Lysozyme Permeability Assay

*P. aeruginosa* PAO1 were grown overnight in LB, then regrown from a 1:100 dilution in fresh media. Mid log phase bacteria (OD_600_ = 0.4–0.6) were harvested, washed once, and resuspended in HEPES buffer (5 mM HEPES at pH 7.2 and 5 mM sodium azide) to an optical absorbance of OD_600_ = 1. Then, 98 μL of bacterial suspension was added to a 96-well plate containing 100 μL of lysozyme solution in PBS. OD_600_ was then measured. The final concentration of lysozyme was 50 μg/mL, and the final OD_600_ was 0.5. Either 2 μL of a chemical compound, or the corresponding solvent, was added to each well. The turbidity of the sample was measured after the lysis process reached equilibrium (as seen by a stabilization in the OD_600_ after mixing) and every 10 s after stabilization for 30 s. Relative values were normalized to PBS as 0% and 99% isopropanol as 100%.

### Nitrocefin Hydrolysis Assay

*P. aeruginosa* PAO1 cells were grown overnight in LB, then regrown (1:100 dilution) in fresh media to an OD_600_ of 0.4–0.5. Cells were centrifuged, washed in PBS, and resuspended to OD_600_ = 0.02 in 20 mM PBS with 1 mM MgCl_2_ at pH 7.2. A volume of 50 μL of the cell suspension was added to a clear, flat-bottom 96-well plate containing 50 μL of PBS with a final concentration of 30 μM nitrocefin and the 2-fold dilution of compound. Plates were incubated at 37 °C in a stationary incubator and read from 0 to 60 min at 10 min intervals at 490 nm to monitor nitrocefin hydrolysis. Reads were normalized using the corresponding no cell control wells.

### DISC_3_(5) Depolarization Assay

*P. aeruginosa* PAO1 were grown overnight in LB, then regrown from a 1:100 dilution in fresh media. Mid log phase bacteria (OD_600_ = 0.4–0.6) were harvested, washed once, and resuspended in HEPES buffer (5 mM HEPES at pH 7.2) to an optical absorbance of OD_600_ = 0.05. Then, 100 μL of 10 mM EDTA was added to 5 mL of resuspended cells for a final concentration of 200 μM EDTA. The bacterial solution was then gently mixed and then let sit for 2 min. Afterward, 5 μL of 0.75 mM DISC_3_(5) was added to the solution for a final concentration of 0.75 μM. Following another gentle mix, the solution was left to incubate in the dark at 37 °C. After incubation, 125 μL of 4 M KCl was added to the cells for a final concentration of 100 mM KCl. Finally, 198 μL of cells and DISC_3_(5) added to a 96-well optical-bottom black plate. Either 2 μL of a chemical compound, or the corresponding solvent, was added to each well. The excitation wavelength was 622 nm, and the emission wavelength was 670 nm. The release of DISC_3_(5) was measured by the increase in fluorescence of DISC_3_(5) for 60 min as a measure of inner membrane depolarization.

### Propidium Iodide

*P. aeruginosa* PAO1 were grown overnight in LB, then regrown from a 1:100 dilution in fresh media for 5 h to an OD_600_ of 0.600. *Pa* cells were harvested (4000 rpm, 25 °C, 10 min), washed, and resuspended in PBS buffer at pH 7.2. Then, 50 μL of a 1.5 mM solution of propidium iodide (PI) was added to the resuspended cells. Following a 60 min incubation, 198 mL of cells and PI added to a 96-well optical-bottom black plate. Either 2 μL of a chemical compound, or the corresponding solvent, was added to each well. The excitation wavelength was 535 nm, and the emission wavelength was 617 nm. The uptake of PI was measured by the increase in fluorescence of PI for 30 min as a measure of inner membrane permeabilization.

### Laurdan GP

An overnight culture of *P. aeruginosa* PAO1 was grown to OD_600_ = 0.4 and diluted to 105 cfu/mL in HEPES buffer, followed by 60 min incubation with Laurdan 2.5 μM at 37 °C in the dark. Following incubation with Laurdan, 198 mL of cells was added to a 96-well plate. Subsequently, 2 mL of compound was added to the wells. The Laurdan fluorescence intensities were measured using a Biotek Synergy H1 spectrophotometer with emission wavelengths of 435 nm and excitation at 490 nm, and the temperature was maintained at 37 °C. Laurdan GP was calculated using the equation GP = (I435 – I490)/(I435 + I490).

### Antagonism Assays

Respective QPC and QAC compounds were serially diluted 2-fold from stock solutions (1.0 mM) to yield 12 test concentrations of 50 μL each, wherein the starting concentration of DMSO was 2.5%. To each well containing 50 μL of the QAC or QPC solution, 50 μL of CCCP (50 μM) and Spd (5 mM) in H_2_O at the designated test concentration was added. Overnight *P. aeruginosa* (PAO1) cultures were regrown to midexponential phase and diluted to ca. 106 cfu/mL in MHB and as determined by optical density recorded at 600 nm (OD_600_). Subsequently, 100 μL were inoculated into each well of a U-bottom 96-well plate (Corning, 351177) containing 100 μL of compound solution. Plates were incubated statically at 37 °C for 24 h upon which wells were evaluated visually for bacterial growth. The MIC was determined as the lowest concentration of compound resulting in no bacterial growth visible to the naked eye, based on the highest value in three independent experiments.

### Whole Genome Sequencing

Genomic DNA extraction, library preparation and Illumina sequencing were performed at the SeqCenter (Pittsburgh, Pennsylvania, USA) using 200 Mbp as the minimum read count per sample. Data was analyzed using breseq (version 0.38.1) as previously described, using the contigs option (−c) when needed.^62^ The following annotated reference genomes were obtained from NCBI and used for the analysis: NC_002516.2 for strain PAO1 and isogenic mutants, and for strains MRSN6220, MRSN6241, MRSN409937, MRSN5524 and isogenic mutants, sequences were obtained from Bioproject PRJNA446057. To identify mutations in isolated resistant mutants, the breseq output was compared to the one obtained from their respective parental (wildtype) strain. Genetic variation also identified in the wildtype parental strain were removed. All the genetic variants are reported in Table S2 (P6P-10,10 resistance associated genetic variations) and S3 (BAC resistance associated genetic variations). Genomic positions and COG functions of genes with mapped genetic variants were obtained in Pseudomonas.com. CLC Genomics Workbench and BLASTn were used to mapped genetic variants to genomic positions.

### Complementation Studies

For genetic complementation, the *smvR* (PA1283) coding sequence containing its predicted native promoter (predicted using SAPPHIRE) was amplified, by PCR, ligated into pUCP30T vector (*Bam*HI and *Eco*RI sites) and plated onto LB agar plates supplemented with gentamycin (60 μg/mL) for selection of transformants (Emory Integrated Genomics Core).^63^ This vector containing *smvR* was confirmed by sequencing. 100 ng of plasmid DNA of empty vector (pUCP30T) and vector containing *smvR* were transformed by electroporation (settings: 25 μF; 200 Ω; 2500 V on a BTX Gemini X2 Electroporation System) into *P. aeruginosa* PAO1 electrocompetent cells prepared as previously described.^64^

### Growth Curves with P6P-10,10

2-fold dilutions of the compound P6P-10,10 were prepared in a flat-bottom 96-well plate (Falcon, 351172). *P. aeruginosa* strains were grown overnight in MHB at 37 °C with shaking (200 ppm) from single colonies grown on LB plates or LB gentamicin 60 μg/mL, when needed. Overnight culture media was supplemented with gentamicin 30 μg/mL for plasmid maintenance, as needed. Cultures were then diluted (1:100 dilution) in fresh MHB (no antibiotic) and grown until mid logarithmic growth phase was reached and then normalized to ca. 10^6^ cfu/mL right before the growth experiment. These fresh bacterial suspensions were used as inoculum in a 1:1 dilution (final cell density ca. 5 × 10^5^ cfu/mL). Plates were incubated at 37 °C with shaking and OD_600_ was measured every 10 min to monitor growth over a 20 h period. Six replicates were performed distributed in two different dates using two different stock solutions of P6P-10,10. Plasmid maintenance was confirmed after each experiment by plating 5 μL aliquots onto LB gentamicin 60 μg/mL plates.

### Hoechst 33342 Dye Accumulation Assay

Hoechst 33342 accumulation assays were performed as previously described.^65^ Briefly, overnight cultures of *P. aeruginosa* in LB were diluted in fresh media and grown until mid logarithmic phase and normalized to an OD_600_ = 0.5. Bacterial cultures were pelleted by centrifugation (10,000*g* × 3 min) and resuspended in PBS. 180 μL of this suspension were used to inoculate a flat-bottom 96-well plate. After two readings, Hoechst 33342 dye was added to final concentration of 2.5 μM in a final volume of 200 μL per well, including a PBS control. Fluorescence was measured from the top of the wells using 360 and 460 nm wavelengths as excitation and emission, respectively. Readings were taken every 2 min for a total of 60 min and normalized to PBS blank controls. Values are reported as fold change in dye accumulation for each strain, accounting for differences in dye at the beginning of experiments. All experiments were performed with at least 3 biological replicates.

## Supporting Information Available

Detailed experimental procedures, supporting figures, plasmids, and genetic details are included (PDF)
