# Supplementary material for: Highly Effective Biocides against Pseudomonas aeruginosa Reveal New Mechanistic Insights Across Gram-Negative Bacteria
Source: ACS Infect Dis. 2024 Oct 23;10(11):3868–79. doi: 10.1021/acsinfecdis.4c00433 (PMC11555683; doi:10.1021/acsinfecdis.4c00433)
Supplement: Supplementary file 1 — id4c00433_si_001.pdf [file id4c00433_si_001.pdf]

Supporting Information

Highly effective biocides against *Pseudomonas aeruginosa* reveal new mechanistic insights across gram-negative bacteria

Christian A. Sanchez[a†], Germán G. Vargas-Cuebas[b†], Marina E. Michaud[a], Ryan A. Allen[a], Kelly R. Morrison-Lewis[a], Shehreen Siddiqui[a], Kevin P.C. Minbiole[c*], and William M. Wuest[a*]

[a]Department of Chemistry, Emory University, Atlanta, GA 30322

[b]Department of Microbiology and Immunology, Emory University, Atlanta, GA 30322

[c]Department of Chemistry, Villanova University, Villanova, PA 1908

*Corresponding authors. Emails: wwuest@emory.edu; kevin.minbiole@villanova.edu

†Co-first authorship.

Table of Contents

1. Materials and Methods S2
2. Supplementary Figures S7
3. Supplementary Tables S14
4. References S19

Materials and Methods

Bacterial strains and growth conditions.

All strains and plasmids are listed in **Table S1**. Bacterial strains were streaked onto lysogeny broth (LB) agar (Sigma-Aldrich, 1102830500) plates and incubated (NuAire, Plymouth, MN) at 37 ºC overnight. Liquid cultures were inoculated with single colonies from plates and incubated for 18-24 hours at 37 ºC with shaking. Media was supplemented with gentamicin (30-60 µg/mL) for vector maintenance in *P. aeruginosa* strains, as needed. *P. aeruginosa* clinical isolates were obtained from the Multidrug-Resistant Organism Repository and Surveillance Network (MRSN).

Growth curves were performed in 96-well flat-bottom plates (Falcon®, 351172) with shaking. Optimal density was measured at a wavelength of 600 nm (OD_{600}) every 10 minutes and growth was monitored over 24 hours. OD_{600} measurements were obtained using a SpectraMax iD3 plate reader (Molecular Devices, United States). Growth curve experiments were performed on different days with independent biological replicates with at least 3 technical replicates per strain/condition.

Minimum Inhibitory Concentration (MIC) Assays

To determine the MIC values, compounds were serially diluted two-fold from stock solutions (1.0 mM) to yield twelve 100 µL test concentrations, wherein the starting concentration of DMSO was 2.5%. Overnight cultures of each strain were diluted to ca. 10^6 CFU/mL in MHB and regrown to mid-exponential phase, as determined by optical density recorded at 600 nm (OD_{600}). All cultures were then diluted again to ca. 10^6 CFU/mL and 100 µL were inoculated into each well of a U-bottom 96-well plate (Falcon, 351177) containing 100 µL of compound solution. Plates were incubated statically at 37 ºC for 72 hours upon which wells were evaluated visually for bacterial growth. The MIC was determined as the lowest concentration of compound resulting in no bacterial growth visible to the naked eye, based on the highest value in three independent experiments. Aqueous DMSO controls were conducted for each strain. Strains of *S. aureus* MSSA (SH1000), *E. coli* (MC4100), *P. aeruginosa* (PAO1), *A. baumannii* (ATCC 17978), CA-MRSA (USA300-0114), and HA-MRSA (ATCC 33591) were grown with shaking at 37 °C overnight from freezer stocks in 5 mL of the indicated media: SH1000, MC4100, USA300-0114, and PAO1 were grown in BD Mueller–Hinton broth (MHB), whereas ATCC 33591 was grown in BD tryptic soy broth (TSB). MRSN isolates were grown in MHB in the same fashion as previously described.

Calculation of IC_{90} Values

To determine the IC90 values for each compound against the clinical isolates, the OD600 for each compound concentration against each strain was recorded. Using Prism 9 (GraphPad software, v. 9.3.1), the IC90 values for each disinfectant compound against each strain were calculated. The OD600 measurements were used as inputs, then normalized to fit 0% (equal to media blank) to 100% (maximum OD600 for each strain).

The analysis was then performed on the normalized data using the dose-response model with a least squares regression fit, wherein outliers (Q=1%) were excluded and no weighting method was applied.

NPN Uptake Assay

P. aeruginosa PAO1 were grown overnight in LB, then regrown from a 1:100 dilution in fresh media for 5 hours to an OD600 of 0.500. Cells were harvested by centrifugation (4000 rpm, 25ºC, 10 min), washed twice with assay buffer (5 mM HEPES, 5 mM glucose, pH 7.2), and resuspended in assay buffer to a final OD600 of 1. Then, 100 µL of washed cells and 100 µL of assay buffer containing 20 µM NPN were together and incubated for 10-30 min. 198 µl of cells and NPN added to a 96-well optical-bottom black plate. Either 2 µL of a chemical compound or the corresponding solvent was added to each well, and fluorescence was immediately monitored at an excitation wavelength of 350 nm and an emission wavelength of 420 nm for 7 minutes at 30 second intervals.

F_{obs} = NPN + cells + compound
$F_{control}$ = NPN + cells
F_b = NPN

NPN uptake = $(F_{obs} – F_B)$—$(F_{control}-F_B)$

20 µM NPN in assay buffer was made from a 5 mM stock of NPN in acetone.

Lysozyme Permeability Assay

P. aeruginosa PAO1 were grown overnight in LB, then regrown from a 1:100 dilution in fresh media. Midlog phase bacteria (OD_{600} = 0.4 – 0.6) were harvested, washed once, and resuspended in HEPES buffer (5 mM HEPES at pH 7.2 and 5 mM sodium azide) to an optical absorbance of OD_{600} = 1. Then, 98 µL of bacterial suspension was added to a 96-well plate containing 100 µL of lysozyme solution in PBS. OD_{600} was then measured. The final concentration of lysozyme was 50 µg/mL, and the final OD_{600} was 0.5. Either 2 µL of a chemical compound, or the corresponding solvent, was added to each well. The turbidity of the sample was measured after the lysis process reached equilibrium (as seen by a stabilization in the OD600 after mixing) and every 10 s after stabilization for 30 s. Relative values were normalized to PBS as 0% and 99% isopropanol as 100%.

Nitrocefin Hydrolysis Assay

P. aeruginosa PAO1 cells were grown overnight in LB, then regrown (1:100 dilution) in fresh media to an OD_{600} of 0.4–0.5. Cells were centrifuged, washed in PBS, and resuspended to OD600 = 0.02 in 20 mM PBS with 1mM MgCl2 at pH 7.2. A volume of 50 µL of the cell suspension was added to a clear, flat-bottom 96-well plate containing 50 µL of PBS with a final concentration of 30 µM nitrocefin and the 2-fold dilution of compound. Plates were incubated at 37 °C in a stationary incubator and read from 0 min to 60 min at

10 min intervals at 490 nm to monitor nitrocefin hydrolysis. Reads were normalized using the corresponding no cell control wells.

DISC$_3$(5) Depolarization Assay

P. aeruginosa PAO1 were grown overnight in LB, then regrown from a 1:100 dilution in fresh media. Midlog phase bacteria (OD_{600} = 0.4 – 0.6) were harvested, washed once, and resuspended in HEPES buffer (5 mM HEPES at pH 7.2) to an optical absorbance of OD_{600} = 0.05. Then, 100 µL of 10 mM EDTA was added to 5 mL of resuspended cells for a final concentration of 200 µM EDTA. The bacterial solution was then gently mixed and then let sit for 2 minutes. Afterwards, 5 µL of 0.75 mM DISC$_3$(5) was added to the solution for a final concentration of 0.75 µM. Following another gentle mix, the solution was left to incubate in the dark at 37ºC. After incubation, 125 µL of 4M KCl was added to the cells for a final concentration of 100 mM KCl. Finally, 198 µL of cells and DISC$_3$(5) added to a 96-well optical-bottom black plate. Either 2 µL of a chemical compound, or the corresponding solvent, was added to each well. The excitation wavelength was 622 nm, and the emission wavelength was 670 nm. The release of DISC$_3$(5) was measured by the increase in fluorescence of DISC$_3$(5) for 60 min as a measure of inner membrane depolarization.

Propidium Iodide

P. aeruginosa PAO1 were grown overnight in LB, then regrown from a 1:100 dilution in fresh media for 5 hours to an OD600 of 0.600. *Pa* cells were harvested (4000 rpm, 25°C, 10 min), washed, and resuspended in PBS buffer at pH 7.2. Then, 50 µL of a 1.5 mM solution of propidium iodide (PI) was added to the resuspended cells. Following a 60-minute incubation, 198 mL of cells and PI added to a 96-well optical-bottom black plate. Either 2 µL of a chemical compound, or the corresponding solvent, was added to each well. The excitation wavelength was 535 nm, and the emission wavelength was 617 nm. The uptake of PI was measured by the increase in fluorescence of PI for 30 min as a measure of inner membrane permeabilization.

Laurdan GP

An overnight culture of *P. aeruginosa* PAO1 was grown to OD600 = 0.4 and diluted to 105 CFU/mL in HEPES buffer, followed by 60 min incubation with Laurdan 2.5 µM at 37 °C in the dark. Following incubation with Laurdan, 198 mL of cells was added to a 96-well plate. Subsequently, 2 mL of compound was added to the wells. The Laurdan fluorescence intensities were measured using a Biotek Synergy H1 spectrophotometer with emission wavelengths of 435 nm and excitation at 490 nm, and the temperature was maintained at 37 °C. Laurdan GP was calculated using the equation GP = (I435 − I490)/(I435 + I490).

Antagonism Assays

Respective QPC and QAC compounds were serially diluted two-fold from stock solutions (1.0 mM) to yield twelve test concentrations of 50 µL each, wherein the starting

concentration of DMSO was 2.5%. To each well containing 50 µL of the QAC or QPC solution, 50 µL of CCCP (50 µM and Spd (5 mM) in H_2O at the designated test concentration was added. Overnight *P. aeruginosa* (PAO1) cultures were regrown to mid-exponential phase and diluted to ca. 106 CFU/mL in MHB and as determined by optical density recorded at 600 nm (OD600). Subsequently, 100 µL were inoculated into each well of a U-bottom 96-well plate (Corning, 351177) containing 100 µL of compound solution. Plates were incubated statically at 37ºC for 24 hours upon which wells were evaluated visually for bacterial growth. The MIC was determined as the lowest concentration of compound resulting in no bacterial growth visible to the naked eye, based on the highest value in three independent experiments.

Whole genome sequencing

Genomic DNA extraction, library preparation and Illumina sequencing were performed at the SeqCenter (Pittsburgh, Pennsylvania, USA) using 200Mbp as the minimum read count per sample. Data was analyzed using breseq (version 0.38.1) as previously described, using the contigs option (-c) when needed[1]. The following annotated reference genomes were obtained from NCBI and used for the analysis: NC_002516.2 for strain PAO1 and isogenic mutants, and for strains MRSN6220, MRSN6241, MRSN409937, MRSN5524 and isogenic mutants, sequences were obtained from Bioproject PRJNA446057. To identify mutations in isolated resistant mutants, the breseq output was compared to the one obtained from their respective parental (wildtype) strain. Genetic variation also identified in the wildtype parental strain were removed. All the genetic variants are reported in **Table S2** (P6P-10,10 resistance associated genetic variations) and **S3** (BAC resistance associated genetic variations). Genomic positions and COG functions of genes with mapped genetic variants were obtained in Pseudomonas.com. CLC Genomics Workbench and BLASTn were used to mapped genetic variants to genomic positions.

Complementation studies

For genetic complementation, the *smvR* (PA1283) coding sequence containing its predicted native promoter (predicted using SAPPHIRE) was amplified, by PCR, ligated into pUCP30T vector (BamHI and EcoRI sites) and plated onto LB agar plates supplemented with gentamycin (60 µg/mL) for selection of transformants (Emory Integrated Genomics Core)[2]. This vector containing *smvR* was confirmed by sequencing. 100 ng of plasmid DNA of empty vector (pUCP30T) and vector containing *smvR* were transformed by electroporation (settings: 25 µF; 200 Ω; 2500 V on a BTX™ Gemini X2 Electroporation System) into *P. aeruginosa* PAO1 electrocompetent cells prepared as previously described[3].

Growth curves with P6P-10,10

Two-fold dilutions of the compound P6P-10,10 were prepared in a flat-bottom 96-well plate (Falcon®, 351172). *P. aeruginosa* strains were grown overnight in MHB at 37 ºC with shaking (200 ppm) from single colonies grown on LB plates or LB gentamicin 60 µg/mL,

when needed. Overnight culture media was supplemented with gentamicin 30 μg/mL for plasmid maintenance, as needed. Cultures were then diluted (1:100 dilution) in fresh MHB (no antibiotic) and grown until mid-logarithmic growth phase was reached and then normalized to ca. 10^6 CFU/mL right before the growth experiment. These fresh bacterial suspensions were used as inoculum in a 1:1 dilution (final cell density ca. 5 x 10^5 CFU/mL). Plates were incubated at 37 ºC with shaking and OD_{600} was measured every 10 minutes to monitor growth over a 20-hour period. Six replicates were performed distributed in two different dates using two different stock solutions of P6P-10,10. Plasmid maintenance was confirmed after each experiment by plating 5 μL aliquots onto LB gentamicin 60 μg/mL plates.

Hoechst 33342 dye accumulation assay

Hoechst 33342 accumulation assays were performed as previously described[4]. Briefly, overnight cultures of *P. aeruginosa* in LB were diluted in fresh media and grown until mid-logarithmic phase and normalized to an OD_{600} = 0.5. Bacterial cultures were pelleted by centrifugation (10,000 g x 3 minutes) and resuspended in PBS. 180 μL of this suspension were used to inoculate a flat-bottom 96-well plate. After two readings, Hoechst 33342 dye was added to final concentration of 2.5 μM in a final volume of 200 μl per well, including a PBS control. Fluorescence was measured from the top of the wells using 360 nm and 460 nm wavelengths as excitation and emission, respectively. Readings were taken every two minutes for a total of 60 minutes. All experiments were performed with at least 3 biological replicates.

Strain	Antibiotic resistance classification
MRSN 3705	Multidrug-resistant resistant clinical isolate from PA7 clade.
MRSN 8141	Multidrug-resistant resistant clinical isolate from PA7 clade.
MRSN 6241	Multidrug-resistant resistant clinical isolate from PA7 clade.
MRSN 6220	Pan-drug resistant isolate.
MRSN 5498	Multidrug-resistant resistant clinical isolate.
MRSN 321	Multidrug-resistant resistant clinical isolate.
MRSN 8915	Multidrug-resistant resistant clinical isolate.
MRSN 2444	Multidrug-resistant resistant clinical isolate.
MRSN 390231	Multidrug-resistant resistant clinical isolate.
MRSN 6695	Multidrug-resistant resistant clinical isolate.
MRSN 1938	Multidrug-resistant resistant clinical isolate.
MRSN 1739	Multidrug-resistant resistant clinical isolate.
MRSN 5508	Multidrug-resistant resistant clinical isolate.
MRSN 8914	Multidrug-resistant resistant clinical isolate.
MRSN 8130	Multidrug-resistant resistant clinical isolate.
MRSN 8912	Multidrug-resistant resistant clinical isolate.
MRSN 5524	Multidrug-resistant resistant clinical isolate.
MRSN 6678	Multidrug-resistant resistant clinical isolate.
MRSN 4841	Multidrug-resistant resistant clinical isolate.
MRSN 5539	Multidrug-resistant resistant clinical isolate.

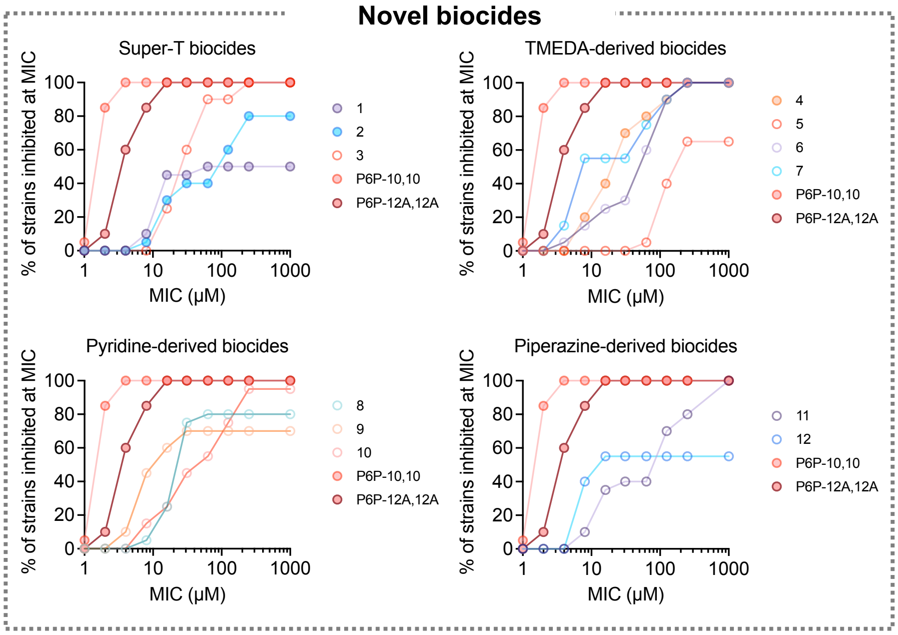

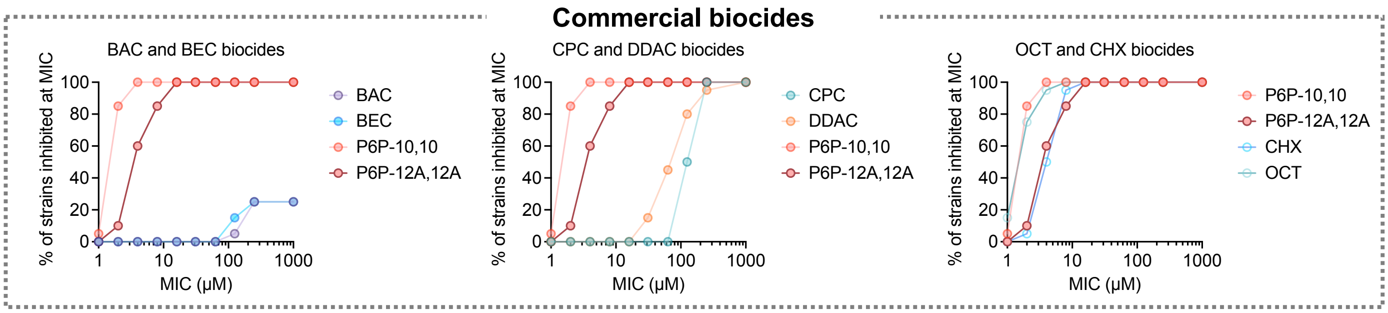

Figure S1. Cumulative inhibition of 20 selected *P. aeruginosa* strains by commercial and novel biocides determined by MIC assays. 20 *P. aeruginosa* clinical isolates with different antibiotic resistance profiles were selected and their susceptibility to novel biocides synthetized by our group and commercial biocides was tested by MIC assay. The cumulative percentage inhibition of the 20 *P. aeruginosa* strains was calculated and plotted against biocide concentration (μM). A trailing growth effect was observed with some of the biocides with specific strains, and IC_{90} values were calculated instead and presented in **Figure 1**.

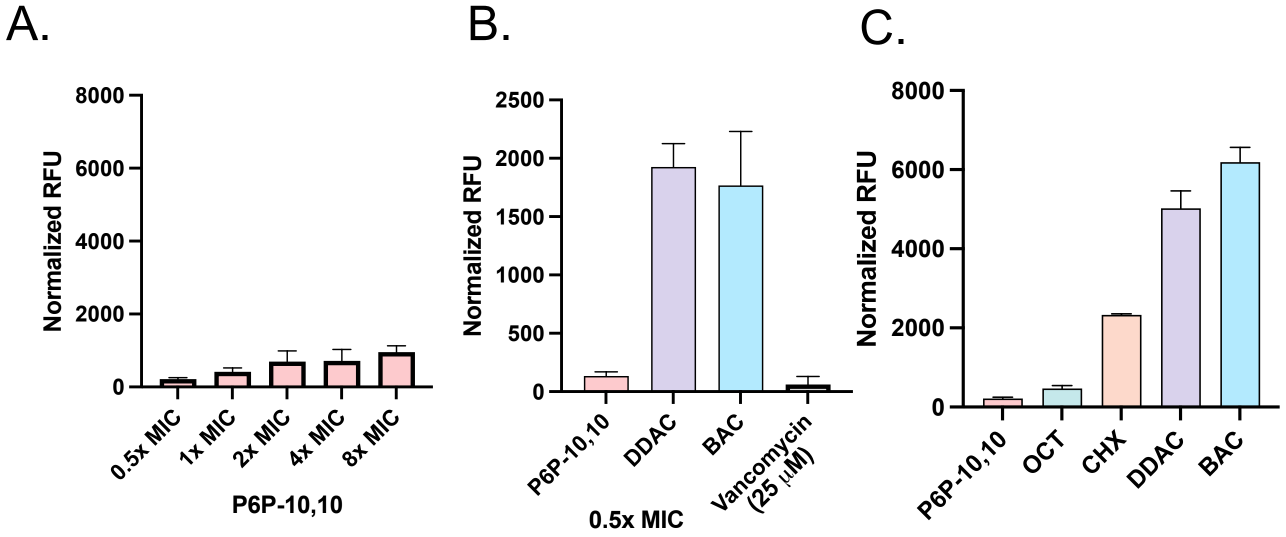

Fig S2. NPN assays varying concentration of cationic biocide and cell density. A) NPN uptake assay with increasing amount of P6P-10,10 to assess outer membrane interactions. B) NPN uptake assay at a 10-fold lower cell density to assess whether inoculum effect influences NPN uptake results with vancomycin as a negative control. C) NPN uptake with P6P-10,10 and BAC at 0.5x, 1x, and 2x relative MIC values show a saturation of outer membrane disruption due to BAC below MIC and no strong effects of higher concentrations of P6P-10,10.

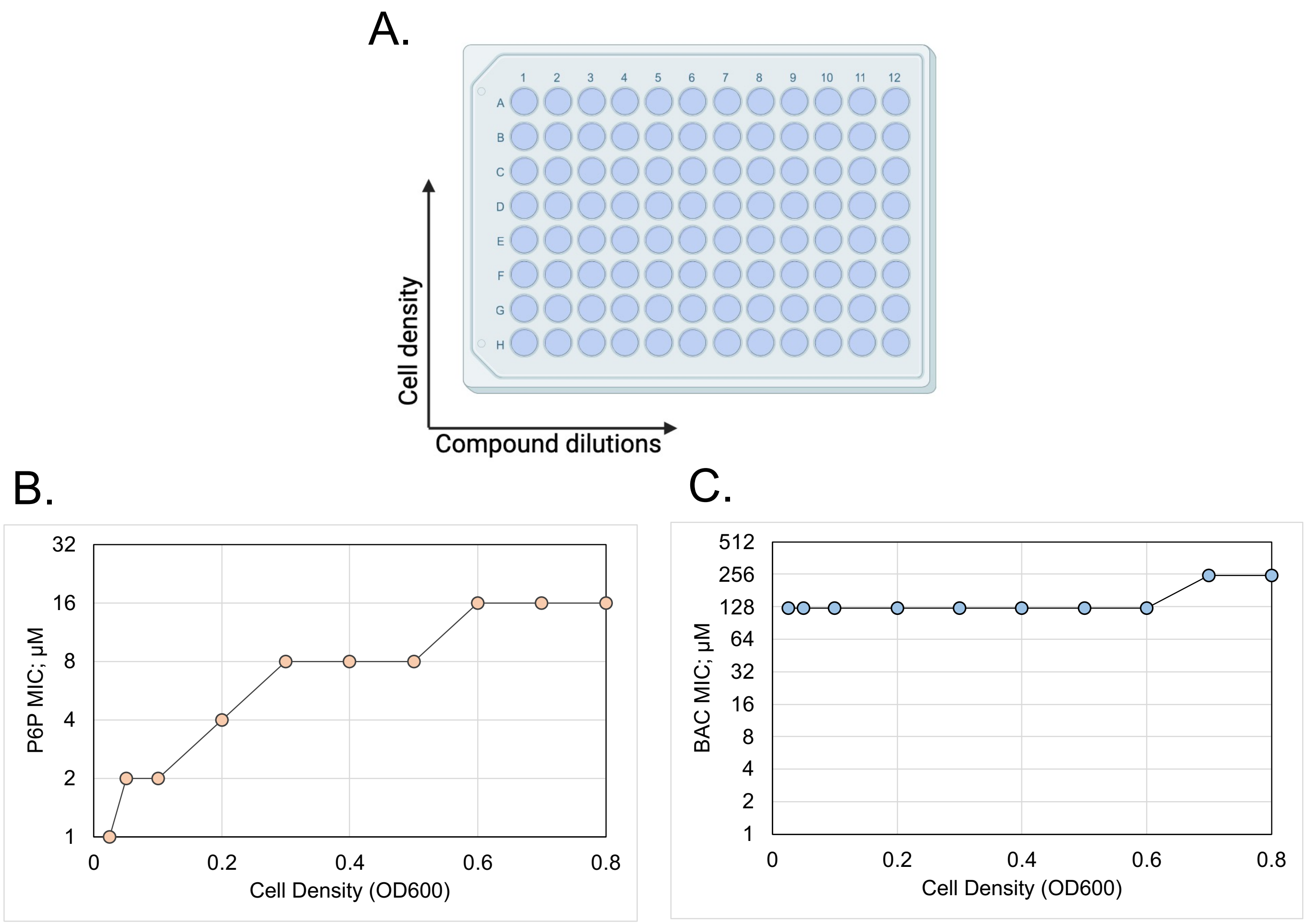

Figure S3: Inoculum effect on P6P-10,10 and BAC antimicrobial activity against *P. aeruginosa* PAO1. A) Schematic of 96-well plate setup to test inoculum effects on P6P-10,10 and BAC minimum inhibitory concentration (MIC). Increasing concentrations of PAO1 cells were exposed to two-fold serial dilutions of the compounds. MIC values were determined after 24 hours of incubation. B) Inoculum effect on P6P-10,10 antimicrobial effectiveness. C) Inoculum effect on BAC antimicrobial effectiveness.

A.

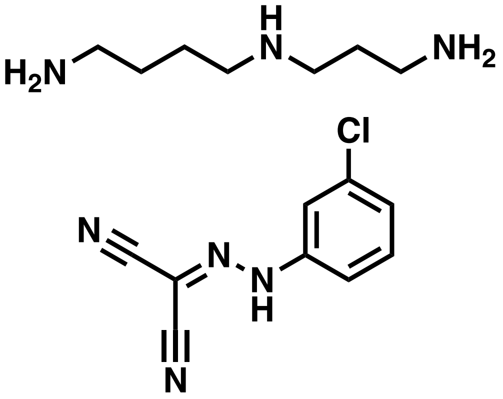

Spermidine (Spd)

Carbonyl cyanide 3-chlorophenyl hydrazone (CCCP)

B.

MIC, µM (± 5mM Spd)

BAC		DDAC		P6P-10,10	
- Spd	+ Spd	- Spd	+ Spd	- Spd	+ Spd
100	300	32	50	2	2

C.

MIC, µM (± 50 µM CCCP)

BAC		DDAC		P6P-10,10	
- CCCP	+ CCCP	- CCCP	+ CCCP	- CCCP	+ CCCP
125	125	32	63	2	40

Figure S4. Spermidine and carbonyl cyanide 3-chlorophenyl hydrazone antagonism assays in *P. aeruginosa* PAO1. A) Chemical structures of spermidine (Spd) and carbonyl cyanide 3-chlorophenyl hydrazone (CCCP). B) MIC data for BAC, DDAC, and P6P-10,10 in the presence of 5mM Spd. C) MIC data for BAC, DDAC, and P6P-10,10 in the presence of 50 µM CCCP.

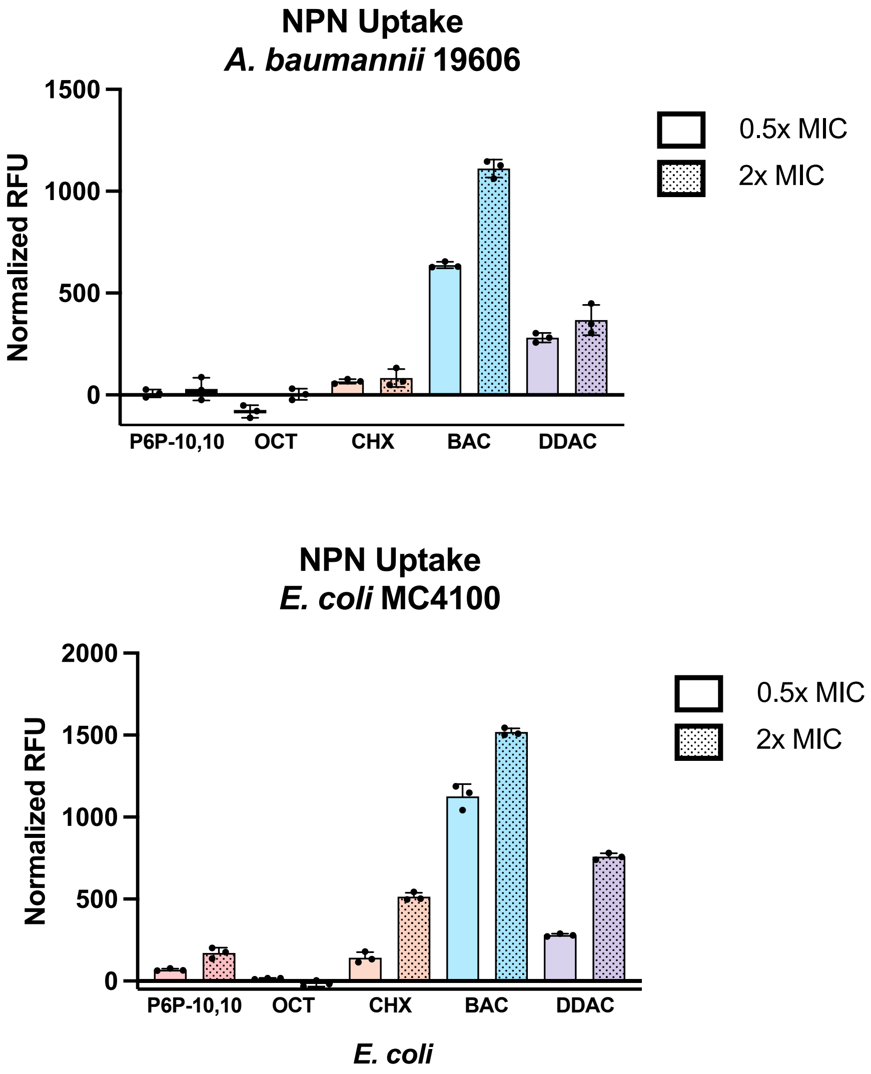

Fig S5. NPN uptake assays in additional gram-negative species. A) NPN uptake was assessed fluorometrically to measure outer membrane perturbation in *A. baumannii* ATCC 19606 at 0.5x and 2x MIC. B) NPN uptake was assessed fluorometrically to measure outer membrane perturbation in *E. coli* MC4100 at 0.5x and 2x MIC.

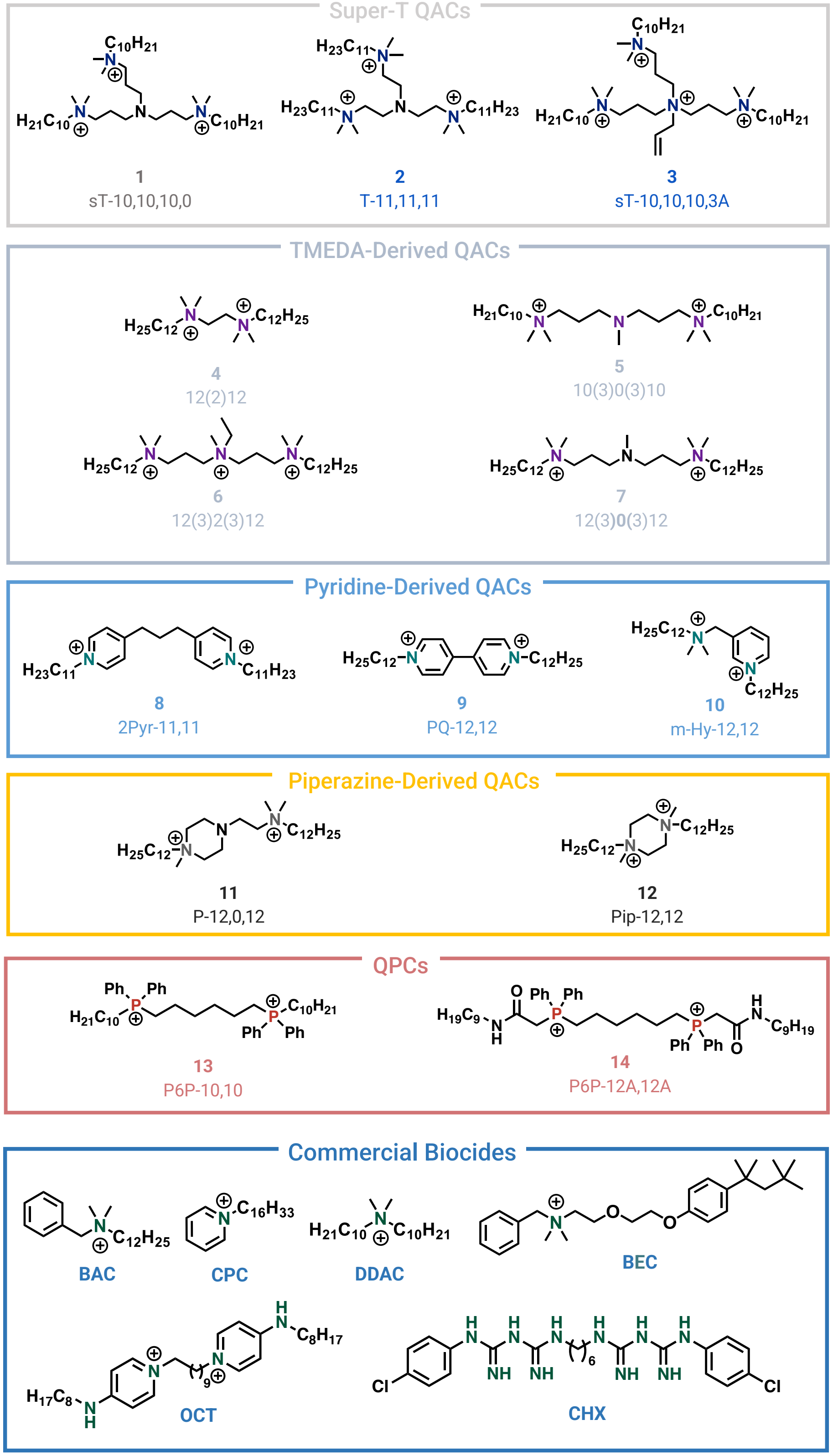

Fig S6. Cationic biocides used in the study grouped by class. Super-T QACs: sT-10,10,10,0 (**1**) , T-11,11,11 (**2**), sT-10,10,10,3A (**3**). TMEDA-Derived QACs: 12(2)12 (**4**), 10(3)0(3)10 (**5**), 12(3)2(3)12 (**6**), 12(3)0(3)12 (**7**). Pyridine-Derived QACs: 2Pyr-11,11 (**8**), PQ-12,12 (**9**), m-Hy-12,12 (**10**). Piperazine-Derived QACs: P-12,0,12 (**11**), Pip-12,12 (**12**). QPCs: P6P-10,10 (**13**), P6P-12A,12A (**14**). Monocationic commercial biocides: benzalkonium chloride (**BAC**), cetylpyridinium chloride (**CPC**), didecyldimethylammonium chloride (**DDAC**), benzethonium chloride (**BEC**). Multicationic commercial biocides: octenidine dichloride (**OCT**), chlorhexidine (**CHX**).

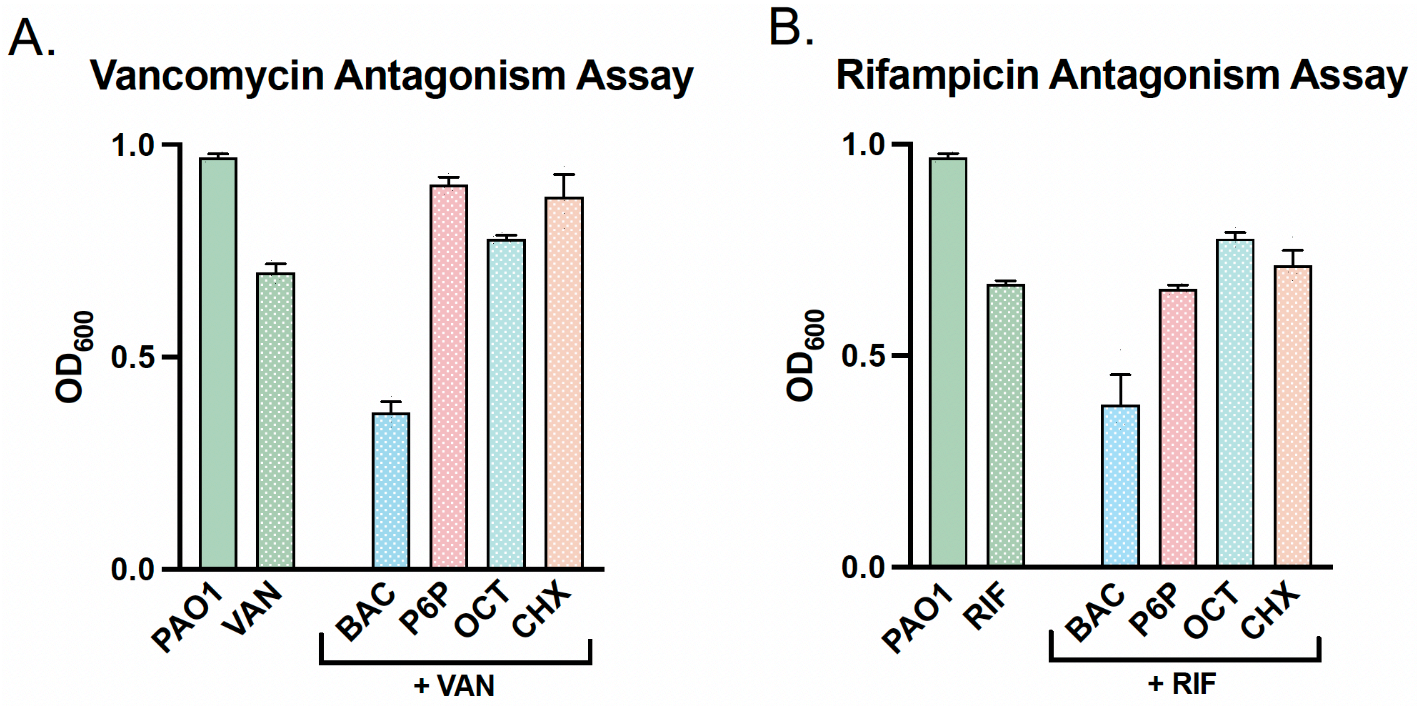

Fig S7. Antagonism assays with gram-positive antibiotics. NPN uptake assay with 0.5x MIC of cationic biocide and A) 16 µM of vancomycin or B) 8 µM of rifampicin. The combination of BAC and antibiotic results in a lower maximum OD_{600} than antibiotic alone, presumably through permeabilizing the membrane and facilitating accumulation. The inner membrane selective biocides had no additive effect on vancomycin and rifampicin.

Table S1: Strains and plasmids used in this study.

Strain name	Description	Source
Strains		
DH5α	*E. coli* cloning strain	Invitrogen
PAO1	*P. aeruginosa* laboratory strain	18
PAO1 BAC^R	Isolated BAC-resistant mutant derived from PAO1.	This study.
PAO1 $P6P^R$	Isolated P6P-resistant mutant derived from PAO1.	This study.
MRSN 6220	Clinical isolate from DoD *P. aeruginosa* diversity panel.	15
MRSN 6220 BAC^R	Isolated BAC-resistant mutant derived from MRSN6220.	This study.
MRSN 6220 $P6P^R$	Isolated P6P-resistant mutant derived from MRSN6220.	This study.
MRSN 6241	Clinical isolate from DoD *P. aeruginosa* diversity panel.	15
MRSN 6241 BAC^R	Isolated BAC-resistant mutant derived from MRSN6241.	This study.
MRSN 6241 $P6P^R$	Isolated P6P-resistant mutant derived from MRSN6241.	This study.
MRSN 409937	Clinical isolate from DoD *P. aeruginosa* diversity panel.	15
MRSN 409937 BAC^R	Isolated BAC-resistant mutant derived from MRSN409937.	This study.
MRSN 409937 $P6P^R$	Isolated P6P-resistant mutant derived from MRSN409937.	This study.
MRSN 5524	Clinical isolate from DoD *P. aeruginosa* diversity panel.	15
MRSN 5524 BAC^R	Isolated BAC-resistant mutant derived from MRSN5524.	This study.
MRSN 5524 $P6P^R$	Isolated P6P-resistant mutant derived from MRSN5524.	This study.
MRSN 3705	Clinical isolate from DoD *P. aeruginosa* diversity panel.	15
MRSN 8141	Clinical isolate from DoD *P. aeruginosa* diversity panel.	15
MRSN 5498	Clinical isolate from DoD *P. aeruginosa* diversity panel.	15
MRSN 321	Clinical isolate from DoD *P. aeruginosa* diversity panel.	15
MRSN 8915	Clinical isolate from DoD *P. aeruginosa* diversity panel.	15
MRSN 2444	Clinical isolate from DoD *P. aeruginosa* diversity panel.	15
MRSN 390231	Clinical isolate from DoD *P. aeruginosa* diversity panel.	15
MRSN 6695	Clinical isolate from DoD *P. aeruginosa* diversity panel.	15
MRSN 1938	Clinical isolate from DoD *P. aeruginosa* diversity panel.	15
MRSN 1739	Clinical isolate from DoD *P. aeruginosa* diversity panel.	15
MRSN 5508	Clinical isolate from DoD *P. aeruginosa* diversity panel.	15
MRSN 8914	Clinical isolate from DoD *P. aeruginosa* diversity panel.	15
MRSN 8130	Clinical isolate from DoD *P. aeruginosa* diversity panel.	15
MRSN 8912	Clinical isolate from DoD *P. aeruginosa* diversity panel.	15
MRSN 6678	Clinical isolate from DoD *P. aeruginosa* diversity panel.	15
MRSN 4841	Clinical isolate from DoD *P. aeruginosa* diversity panel.	15
MRSN 5539	Clinical isolate from DoD *P. aeruginosa* diversity panel.	15
Plasmids		
pUCP30T	Parental plasmid used for *trans*-complementation (Tet^R)	25
pUCP30T::*smvR*	pUCP30T containing *smvR* with its predicted native promoter	This study.

Table S2: Genetic variants identified in P6P-resistant strains.

gene	position	mutation	PAO1 locus tag	description
PAO1 P6P-R				
[*smvR*]–[PA1284]	1,394,696	Δ146 bp	PA1283	[*smvR*], [PA1284]
pssA ←	5,271,885	A139V (GCG→GTG)	PA4693	phosphatidylserine synthase
MRSN6220 P6P-R				
DY941_RS06195 ←	18,779	$(ATCAGCGCCGCC)_{2\rightarrow1}$	PA1283	TetR/AcrR family transcriptional regulator
DY941_RS06850 ←	79,961	N187S (AAC→AGC)	PA1157	winged helix-turn-helix domain-containing protein
DY941_RS09310 →	223,123	E128* (GAG→TAG)	PA0663	hypothetical protein
pgsA ←	60,954	A51T (GCC→ACC)	PA2584	CDP-diacylglycerol--glycerol-3-phosphate 3-phosphatidyltransferase
DY941_RS23730 ←	30,560	Y26H (TAC→CAC)	PA2262	MFS transporter
MRSN6241 P6P-R				
DY940_RS05995 →	317,974	L12* (TTG→TAG)	*PA1283 (smvR)*	*smvR* TetR/AcrR family transcriptional regulator
MRSN409937 P6P-R				
[DY952_RS03800]	338,867	Δ99 bp	*PA1283 (smvR)*	*smvR*
tatC ←	66,282	L61F (CTT→TTT)	*PA5070*	twin-arginine translocase subunit TatC
intergenic	15,636	$(A)_{9\rightarrow8}$	*PA3690 / PA3691*	DUF4398 domain-containing protein/heavy metal translocating P-type ATPase
mexY ←	37,600	2 bp→CA	*PA2018*	multidrug efflux RND transporter permease subunit MexY
mexY ←	37,603	3 bp→A	*PA2018*	multidrug efflux RND transporter permease subunit MexY
MRSN5524 P6P-R				
DY943_RS12890 ←	17,893	$(ATCAGCGCCGCC)_{2\rightarrow3}$	*PA1283*	smvR
mexY →	76,038	Δ1 bp	*PA2018*	multidrug efflux RND transporter permease subunit MexY

Table S3: Genetic variants identified in BAC-resistant strains.

gene	position	mutation	PAO1 locus tag	description
PAO1 BAC-R				
cupC2 →	1,074,592	$(C)_{6\to5}$	PA0993	chaperone CupC2
aprD →	1,351,186	Y147C (TAC→TGC)	PA1246	alkaline protease secretion protein AprD
PA2867 →	3,220,511	G302S (GGC→AGC)	PA2867	probable chemotaxis transducer
mutS →	4,055,923	N467D (AAC→GAC)	PA3620	DNA mismatch repair protein MutS
PA4667 →	5,235,708	coding (712/1773 nt)	PA4667	hypothetical protein
MRSN6220 BAC-R				
DY941_RS02760 ←	17,679	$(T)_{9\to8}$	PA5133	murein hydrolase activator EnvC
DY941_RS03340 ←	4,227	G340G (GGC→GGT)	PA5021	potassium/proton antiporter
dipA ←	9,293	Δ13 bp	PA5017	phosphodiesterase DipA
DY941_RS05315 ←	31,937	Y16Y (TAC→TAT)	PA1463	chemotaxis protein CheW
DY941_RS05340 ←	37,996	D39D (GAC→GAT)<br>G164G (GGA→GGG)	PA1458	chemotaxis protein CheA
lasR ←	66,473		PA1430	transcriptional regulator LasR
DY941_RS06180 ←	15,375	V219I (GTC→ATC)	PA1286	MFS transporter
phoQ ←	56,267	L312P (CTG→CCG)	PA1180	two-component system sensor histidine kinase PhoQ
DY941_RS07355 →	30,120	R192C (CGT→TGT)	PA0291	OprD family porin
intergenic	83,144	C→T	PA0373	insulinase family protein/signal recognition particle-docking protein FtsY
DY941_RS08390 →	50,598	R103W (CGG→TGG)	PA0483	GNAT family N-acetyltransferase
DY941_RS11375 →	22,861	S10S (AGC→AGT)	PA3937	taurine ABC transporter ATP-binding protein
DY941_RS11615 →	70,312	S116S (AGC→AGT)		regulatory protein GemA
DY941_RS11900 ←	122,033	F68L (TTC→CTC)	PA3886	TIGR01459 family HAD-type hydrolase
intergenic	22,126	A→G	PA3828/29 intergenic	alpha/beta hydrolase/LPS export ABC transporter permease LptF
DY941_RS13070 →	59,888	V71A (GTT→GCT)	PA4440	alpha/beta hydrolase
murD →	14,515	V313V (GTA→GTG)	PA4414	UDP-N-acetylmuramoyl-L-alanine--D-glutamate ligase
intergenic	2,853	C→T	PA2042	APC family permease/serine/threonine transporter SstT
ligD →	120,803	D298G (GAC→GGC)	PA2138	DNA ligase D
tagH ←	9,833	$(GGCTGT)_{10\to7}$	PA0081	type VI secretion system-associated FHA domain protein TagH
DY941_RS16375 ←	56,939	S213S (AGC→AGT)	PA0780	AraC family transcriptional regulator
DY941_RS16525 →	86,312	G71S (GGC→AGC)	PA0751	AbrB family transcriptional regulator
intergenic	11,056	G→A	PA3300/01	long-chain-fatty-acid--CoA ligase FadD2/alpha/beta hydrolase
DY941_RS18410 ←	26,646	Δ2 bp	PA0011	lysophospholipid acyltransferase
DY941_RS19265 ←	208,282	$(CCG)_{2\to3}$	PA5418	sarcosine oxidase subunit alpha
DY941_RS20300 ←	3,314	V117A (GTG→GCG)		hypothetical protein
cdsA ←	19,282	P132L (CCG→CTG)	PA3651	phosphatidate cytidylyltransferase
rpsB ← / → *map*	23,774	T→C	PA3656	30S ribosomal protein S2/type I methionyl aminopeptidase
pheT →	23,793	Y658C (TAC→TGC)	PA2739	phenylalanine--tRNA ligase subunit beta
DY941_RS22510 ←	47,540	R114G (AGA→GGA)		inovirus Gp2 family protein
intergenic	26,063	$(G)_{6\to7}$	PA2379/80	hypothetical protein/(2Fe-2S)-binding protein
DY941_RS23690 ←	20,551	Δ1 bp	PA2270	TetR family transcriptional regulator
mutS →	45,820	T112P (ACC→CCC)	PA3620	DNA mismatch repair protein MutS
intergenic	7,090	$(C)_{6\to7}$	PA4028/29	hypothetical protein/DedA family protein
DY941_RS25610 →	50,523	V38I (GTC→ATC)	PA4076	hypothetical protein
DY941_RS25645 ←	1,754	N719S (AAC→AGC)	PA4082	filamentous hemagglutinin N-terminal domain-containing protein
DY941_RS27435 ←	2,249	A189T (GCC→ACC)	PA1015	IclR family transcriptional regulator
DY941_RS27520 →	19,818	M1M (ATG→GTG) †	PA1028/29	DUF2384 domain-containing protein
DY941_RS28400 ←	25,649	R54C (CGC→TGC)	PA4173	RidA family protein
MRSN6241 BAC-R				
DY940_RS01155 →	88,254	Δ15 bp	*PA0011*	lysophospholipid acyltransferase
intergenic	22,050	$(TCTCCTG)_{10\to9}$	intergenic	Hpt domain-containing protein/type 1 fimbrial protein
DY940_RS07645 ←	181,779	V389A (GTG→GCG)	*PA1561*	methyl-accepting chemotaxis protein
DY940_RS24055 ←	79,343	$(TTCTCGATG)_{3\to2}$	*PA4670*	ribose-phosphate pyrophosphokinase
DY940_RS27745 →	50,110	T225P (ACC→CCC)	*PA5332*	exodeoxyribonuclease III
MRSN409937 BAC-R				
tpbB ←	159,229	A196T (GCG→ACG)	*PA1120*	diguanylate cyclase TpbB
DY952_RS25190 ←	100,076	H229R (CAT→CGT)	*PA0011*	lysophospholipid acyltransferase
algB →	78,955	P205S (CCG→TCG)	*PA5483*	sigma-54-dependent response regulator transcription factor AlgB
DY952_RS29250 →	59,032	A21V (GCG→GTG)	*PA2270*	TetR family transcriptional regulator
MRSN5524 BAC-R				
DY943_RS04370	2,749	IS5	PA3450	glycosyltransferase family 4 protein

Table S4: BAC and P6P-10,10 MIC values in resistant mutant and parental strains.

	MIC (µM)	
**Strain**	**BAC**	**P6P-10,10**
PAO1	125	2
BAC^R	250-500	2-4
P6P^R	125-250	8-16
MRSN 6220	125	2
6220 BAC^R	250	2
6220 P6P^R	125	16
MRSN 6241	250	2
6241 BAC^R	250	2-4
6241 P6P^R	125-250	8-16
MRSN 409937	125	2
409937 BAC^R	125-250	1-4
409937 P6P^R	125	8-16
MRSN 5524	500	2
5524 BAC^R	500	2-4
5524 P6P^R	500	8-16

Table S5. Susceptibility of the *P. aeruginosa* clinical isolates to a panel of 14 antibiotics, 4 commercial QACs and 2 multicationic biocides, and 14 of our previously reported cationic disinfectant compounds.

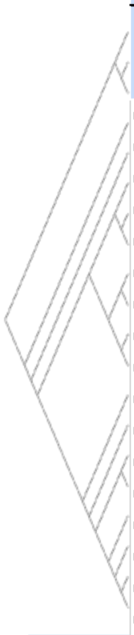

		Antbiotic Suceptibiltiy													
		Anitbiotic Classes													
		Aminoglycoside			Cephalosporin			Beta-lactam				Carbapenem		Quinolone	
**PA7 clade**	**exoU+/exoS+**	**AMK**	**GEN**	**TOB**	**ATM**	**CAZ**	**FEP**	**TZP**	**TIM**	**CZA**	**C/T**	**IPM**	**MEM**	**CIP**	**LVX**
**MRSN 3705**	-	S	S	S	R	R	R	R	R	S	S	S	S	R	R
**MRSN 8141**	-	S	R	R	R	R	R	R	R	R	R	R	R	R	R
**MRSN 6241**	-	S	R	R	R	R	R	R	R	R	R	R	R	R	R
MRSN 6220	S	**R**	**R**	**R**	**R**	**R**	**R**	**R**	**R**	**R**	**R**	**R**	**R**	**R**	**R**
MRSN 5498	S	S	R	R	R	S	R	R	R	S	R	R	R	R	R
MRSN 321	S	S	S	S	R	R	R	R	R	S	S	R	R	S	S
MRSN 8915	S	S	R	R	S	S	S	R	R	S	S	R	R	R	R
MRSN 2444	S	R	R	R	R	R	R	S	R	S	S	R	R	R	R
MRSN 390231	S	S	S	S	R	S	S	R	R	S	S	S	S	R	S
MRSN 6695	S	S	S	S	R	R	R	R	S	S	S	R	R	R	R
MRSN 1938	S	S	R	R	S	S	S	S	S	S	S	R	R	R	R
MRSN 1739	S/U	S	R	R	R	R	R	R	R	S	S	R	R	R	R
MRSN 5508	S	S	S	S	R	R	R	R	R	R	S	R	R	S	S
MRSN 8914	S	R	R	R	R	R	R	R	R	S	S	R	R	R	R
MRSN 8130	U	R	S	R	R	S	R	R	R	S	S	R	R	R	R
MRSN 8912	U	S	R	R	R	S	R	R	R	S	S	R	R	R	R
MRSN 5524	U	S	R	R	R	S	R	S	R	S	S	R	R	R	R
MRSN 6678	U	S	R	R	R	R	R	R	R	R	S	R	R	R	R
MRSN 4841	U	S	R	S	R	R	R	R	R	R	S	S	S	R	R
MRSN 5539	U	S	R	S	R	R	R	R	R	S	S	R	R	R	R

	Disinfectant Suceptibility (IC_{90}, μM)																			
	Commercial Biocides						Synthesized Cationic Disinfectant Compounds													
	Monocationic				Multicationic		Super-T			TMEDA-Derived				Pyridine-Derived			Piperazine		QPC	
**PA7 clade**	**BAC**	**BEC**	**CPC**	**DDAC**	**CHX**	**OCT**	**1**	**2**	**3**	**4**	**5**	**6**	**7**	**8**	**9**	**10**	**11**	**12**	**13**	**14**
**MRSN 3705**	>250	>250	>250	>250	16	2	>250	>250	>250	>250	>250	>250	>250	>250	>250	>250	>250	>250	4	4
**MRSN 8141**	>250	>250	>250	>250	8	2	19	>250	227	>250	39	79	189	46	>250	>250	>250	>250	4	4
**MRSN 6241**	**>250**	**>250**	**>250**	**>250**	**8**	**1**	**>250**	**>250**	**>250**	**>250**	**>250**	**>250**	**>250**	**>250**	**>250**	**>250**	**>250**	**>250**	4	4
MRSN 6220	188	237	148	229	8	2	8	>250	>250	>250	86	70	116	8	>250	>250	>250	>250	2	2
MRSN 5498	>250	>250	>250	232	4	2	>250	>250	>250	>250	>250	210	>250	>250	>250	>250	>250	>250	2	4
MRSN 321	>250	>250	190	>250	8	2	>250	>250	>250	>250	>250	>250	>250	>250	>250	>250	>250	>250	2	2
MRSN 8915	212	>250	181	2	8	4	7	104	17	156	23	8	18	32	19	39	9	45	2	8
MRSN 2444	>250	>250	130	>250	4	1	8	17	8	156	7	8	16	7	>250	32	77	39	2	8
MRSN 390231	>250	185	>250	67	2	2	6	55	51	125	4	4	18	3	4	0	10	8	1	2
MRSN 6695	>250	>250	136	85	8	4	>250	>250	>250	>250	>250	>250	>250	>250	16	74	8	32	2	16
MRSN 1938	>250	126	110	60	4	2	7	16	8	130	7	16	35	14	7	12	21	9	2	8
MRSN 1739	>250	>250	129	133	4	4	8	>250	50	133	16	13	18	14	4	31	8	8	2	16
MRSN 5508	>250	193	132	70	8	8	>250	>250	>250	>250	>250	>250	>250	>250	>250	206	192	44	2	8
MRSN 8914	>250	>250	135	>250	4	1	>250	>250	>250	152	233	>250	>250	>250	>250	>250	>250	>250	2	4
MRSN 8130	>250	>250	139	>250	8	4	8	>250	>250	166	>250	>250	>250	>250	>250	163	>250	>250	2	8
MRSN 8912	>250	126	170	101	4	2	>250	164	144	165	16	56	33	>250	8	45	15	14	2	4
MRSN 5524	>250	>250	175	>250	4	2	8	>250	>250	>250	>250	>250	>250	>250	>250	>250	>250	>250	2	16
MRSN 6678	>250	>250	>250	>250	4	2	>250	>250	>250	141	249	135	>250	>250	>250	>250	>250	>250	2	4
MRSN 4841	>250	>250	>250	243	8	2	9	>250	157	>250	139	177	47	11	>250	>250	>250	>250	2	4
MRSN 5539	>250	>250	162	>250	4	2	8	106	26	204	8	8	16	7	15	120	19	17	2	4

Super-T QACs: sT-10,10,10,0 (**1**) , T-11,11,11 (**2**), sT-10,10,10,3A (**3**). TMEDA-Derived QACs: 12(2)12 (**4**), 10(3)0(3)10 (**5**), 12(3)2(3)12 (**6**), 12(3)0(3)12 (**7**). Pyridine-Derived QACs: 2Pyr-11,11 (**8**), PQ-12,12 (**9**), m-Hy-12,12 (**10**). Piperazine-Derived QACs: P-12,0,12 (**11**), Pip-12,12 (**12**). QPCs: P6P-10,10 (**13**), P6P-12A,12A (**14**). Monocationic commercial biocides: benzalkonium chloride (**BAC**), cetylpyridinium chloride (**CPC**), didecyldimethyl ammonium chloride (**DDAC**), benzethonium chloride (**BEC**). Multicationic commercial biocides: octenidine dichloride (**OCT**), chlorhexidine (**CHX**)

References

1. Deatherage, D. E.; Barrick, J. E. Identification of mutations in laboratory-evolved microbes from next-generation sequencing data using breseq. *Methods Mol Biol* **2014**, *1151*, 165-188. DOI: 10.1007/978-1-4939-0554-6_12

2. Coppens, L.; Lavigne, R. SAPPHIRE: a neural network based classifier for sigma70 promoter prediction in Pseudomonas. *BMC Bioinformatics* **2020**, *21* (1), 415. DOI: 10.1186/s12859-020-03730-z

3. Choi, K. H.; Kumar, A.; Schweizer, H. P. A 10-min method for preparation of highly electrocompetent Pseudomonas aeruginosa cells: application for DNA fragment transfer between chromosomes and plasmid transformation. *J Microbiol Methods* **2006**, *64* (3), 391-397. DOI: 10.1016/j.mimet.2005.06.001

4. Sobhanipoor, M. H.; Ahmadrajabi, R.; Nave, H. H.; Saffari, F. Determination of efflux activity in Enterococci by Hoechst accumulation assay and the role of zinc oxide nanoparticles in inhibition of this activity. *BMC Microbiol* **2022**, *22* (1), 195. DOI: 10.1186/s12866-022-02595-x
